# Global population exposure to landscape fire air pollution from 2000 to 2019

**DOI:** 10.1038/s41586-023-06398-6

**Published:** 2023-09-20

**Authors:** Rongbin Xu, Tingting Ye, Xu Yue, Zhengyu Yang, Wenhua Yu, Yiwen Zhang, Michelle L. Bell, Lidia Morawska, Pei Yu, Yuxi Zhang, Yao Wu, Yanming Liu, Fay Johnston, Yadong Lei, Michael J. Abramson, Yuming Guo, Shanshan Li

**Affiliations:** 1 Climate, Air Quality Research Unit, School of Public Health and Preventive Medicine, Monash University, Melbourne, Victoria Australia; 2 Jiangsu Key Laboratory of Atmospheric Environment Monitoring and Pollution Control, Collaborative Innovation Center of Atmospheric Environment and Equipment Technology, Joint International Research Laboratory of Climate and Environment Change, School of Environmental Science and Engineering, Nanjing University of Information Science and Technology, Nanjing, China; 3 School of the Environment, Yale University, New Haven, CT USA; 4 International Laboratory for Air Quality and Health, Queensland University of Technology, Brisbane, Queensland Australia; 5 Menzies Institute for Medical Research, University of Tasmania, Hobart, Tasmania Australia; 6 State Key Laboratory of Severe Weather and Key Laboratory of Atmospheric Chemistry of CMA, Chinese Academy of Meteorological Sciences, Beijing, China

**Keywords:** Environmental impact, Risk factors, Environmental health

## Abstract

Wildfires are thought to be increasing in severity and frequency as a result of climate change^1–5^. Air pollution from landscape fires can negatively affect human health^4–6^, but human exposure to landscape fire-sourced (LFS) air pollution has not been well characterized at the global scale^7–23^. Here, we estimate global daily LFS outdoor fine particulate matter (PM_2.5_) and surface ozone concentrations at 0.25° × 0.25° resolution during the period 2000–2019 with the help of machine learning and chemical transport models. We found that overall population-weighted average LFS PM_2.5_ and ozone concentrations were 2.5 µg m^−3^ (6.1% of all-source PM_2.5_) and 3.2 µg m^−3^ (3.6% of all-source ozone), respectively, in 2010–2019, with a slight increase for PM_2.5_, but not for ozone, compared with 2000–2009. Central Africa, Southeast Asia, South America and Siberia experienced the highest LFS PM_2.5_ and ozone concentrations. The concentrations of LFS PM_2.5_ and ozone were about four times higher in low-income countries than in high-income countries. During the period 2010–2019, 2.18 billion people were exposed to at least 1 day of substantial LFS air pollution per year, with each person in the world having, on average, 9.9 days of exposure per year. These two metrics increased by 6.8% and 2.1%, respectively, compared with 2000–2009. Overall, we find that the global population is increasingly exposed to LFS air pollution, with socioeconomic disparities.

## Abstract (web summary)

The global population is increasingly exposed to daily landscape fire-sourced air pollution but there are socioeconomic disparities, with this pollution four times higher in low-income countries than in high-income countries during the period 2000–2019.

## Main

The term landscape fires refers to any fires burning in natural and cultural landscapes, for example natural and planted forest, shrub, grass, pastures, agricultural lands and peri-urban areas^24^. It includes both planned or controlled fires (for example, prescribed burns, agricultural fires) and wildfires (defined as uncontrolled or unplanned fires burning in wildland vegetation^25^). There is evidence that wildfires are increasingly frequent and severe as a result of climate change^1–5^. Compared with the direct exposure to the flames and heat of landscape fires, the exposure to air pollution caused by landscape fire smoke travelling hundreds, and sometimes even thousands, of kilometres^4^ can affect much larger populations, and cause much larger public health risks^6^. Mapping and tracking population exposure to landscape fire-sourced (LFS) air pollution (mainly including particulate matter with a diameter of 2.5 µm or less (PM_2.5_) and ozone (O_3_)) are essential for monitoring and managing the health impacts of such fires, implementing targeted prevention and interventions, and strengthening arguments for mitigation of climate change.

However, there are a lack of accurate daily fire-sourced air pollution data with complete spatiotemporal coverage across the globe. Wildfires often mainly threaten suburban, rural and remote areas where there are few or no air quality monitoring stations^4^. In many low-income countries, there are no air quality monitoring stations even in urban areas. Therefore, the data gap cannot be addressed by using air quality monitoring stations alone.

Our previous studies have estimated the daily fire-sourced PM_2.5_ for Brazil^7^ and 749 worldwide locations^8^ during the period 2000–2016. Many studies also estimated fire-related PM_2.5_ in the USA^9–18^ and Europe^19,20^ using various approaches (for example, chemical transport models, satellite-based fire smoke plume, machine learning). However, there are still a lack of data in many other regions, particularly sub-Saharan Africa and Southeast Asia where landscape fires are frequent^21^.Two early studies attempted to address the data gap at a global scale using chemical transport models; they estimated global daily fire-sourced PM_2.5_ for 1997–2006^22^ and 2016–2019^23^. However, the accuracy of chemical transport model outputs could be problematic without calibration against observations of air quality monitoring stations^16^, and these two global studies could not assess the long-term trend of fire-sourced PM_2.5_ given their short study periods. Furthermore, to our knowledge, no previous study has estimated global LFS O_3_. This important fire-related pollutant has been estimated only for the USA using chemical transport models without calibration against station observations^9,13^. Last but not the least, all these previous studies focused mainly on data generation or health impact assessment; little attention has been paid to population exposure assessment.

This study estimated the daily fire-sourced PM_2.5_ and O_3_ concentrations at 0.25° × 0.25° (about 28 km × 28 km at the equator) spatial resolution across the globe from 2000 to 2019. Through linking the dataset with global population distribution data, we aimed to perform a comprehensive assessment of global population exposures to fire-sourced PM_2.5_ and O_3_ during the period 2000–2019.

## Data validation

As detailed in Methods, Extended Data and Supplementary Information, we validated our estimated all-source and fire-sourced PM_2.5_ and O_3_ in several ways.

The spatial tenfold cross-validation (CV) (that is, by dividing all stations into ten approximately equal subsets, then performing validation of the model estimates on each subset for the model trained in the remaining nine subsets) demonstrated our machine learning models’ high level of accuracy in estimating both all-source daily average PM_2.5_ (*R*^2^ = 0.89, root mean squared error (RMSE) = 9.24 µg m^−3^) and all-source daily maximum 8 h O_3_ (*R*^2^ = 0.80, RMSE = 19.24 µg m^−3^) in new locations not in the training data. As a further test of our model’s ability to generalize to regions far from available training stations, we clustered globally available PM_2.5_ and O_3_ stations into 75 and 99 contiguous clusters, respectively, and used leave-one-out CV to evaluate model performance on each cluster as it was temporarily excluded from model training. As expected, performance was lower than the spatial tenfold CV. In clusters in which the model was not trained, the model estimates explained 69% and 67% of the overall variations in all-source PM_2.5_ and O_3_, respectively, and 41% and 52% of local temporal daily variations (that is, after excluding variations across stations and between years) of all-source PM_2.5_ and O_3_, respectively. This performance, however, was still much higher than the performance of the uncalibrated raw GEOS-Chem outputs, suggesting that our models can predict the daily all-source PM_2.5_ and O_3_ in large remote areas with no training data with an accuracy much higher than that of the raw GEOS-Chem outputs alone.

Notably, in most regions of the world, we are able to evaluate our model performance in predicting variation only in all-source, but not fire-sourced, PM_2.5_ and O_3_. We made two further efforts to validate our estimated fire-sourced PM_2.5_ and O_3_ in some regions.

First, under a straightforward hypothesis that the station-observed PM_2.5_ and O_3_ during wildfire events are caused mainly by wildfire smoke, we chose ten large wildfire events in Australia, the USA, Chile, Portugal and South Africa to validate our estimated all-source and fire-sourced PM_2.5_ and O_3_. For each wildfire event, we chose the most affected monitoring station (that is, the nearby station showing the largest increase in observed concentrations during the wildfire event, compared with the pre-wildfire period) as the validation target. During the wildfire event and up to 60 days before and after the event, the observed daily all-source PM_2.5_ or O_3_ from the most affected station showed good agreement with our estimated daily all-source PM_2.5_ (*R*^2^ = 0.64 on average across events) and O_3_ (*R*^2^ = 0.78) based on a model trained in stations excluding all nearby stations, although our estimates tended to substantially understate PM_2.5_ concentrations during some extreme PM_2.5_ periods. Furthermore, we observed an expected increase in the estimated concentrations and proportions (among all sources) of fire-sourced PM_2.5_ and O_3_ during the selected wildfire events, compared with the pre-wildfire period, suggesting that our models can reasonably capture the wildfires’ impacts on the daily PM_2.5_ and O_3_ concentrations.

Second, we compared our estimated fire-sourced PM_2.5_ with the smoke PM_2.5_ (that is, PM_2.5_ concentrations attributable to fire smoke overhead detected by satellite images) estimated by Childs et al.^17^ in the contiguous USA, and found a high agreement (Pearson correlation coefficient *r* = 0.88). When further validated against the smoke PM_2.5_ observed by 2,147 PurpleAir stations that were neither in our training data nor in those of Childs et al.^17^, our estimated fire-sourced PM_2.5_ (*R*^2^ = 0.51, RMSE = 11.76 µg m^−3^) showed lower accuracy than the estimated smoke PM_2.5_ of Childs et al.^17^ (*R*^2^ = 0.66, RMSE = 10.46 µg m^−3^), perhaps as a result of our attempts to build a globally generalizable model. However, our performance was still much greater than the accuracy of the fire-sourced PM_2.5_ from raw GEOS-Chem outputs (*R*^2^ = 0.18, RMSE = 22.96 µg m^−3^).

On the basis of our validated data, the global population exposures to fire-sourced PM_2.5_ and O_3_ were described as follows.

## Fire-sourced PM_2.5_ and O_3_ concentrations

**Fig. 1:**
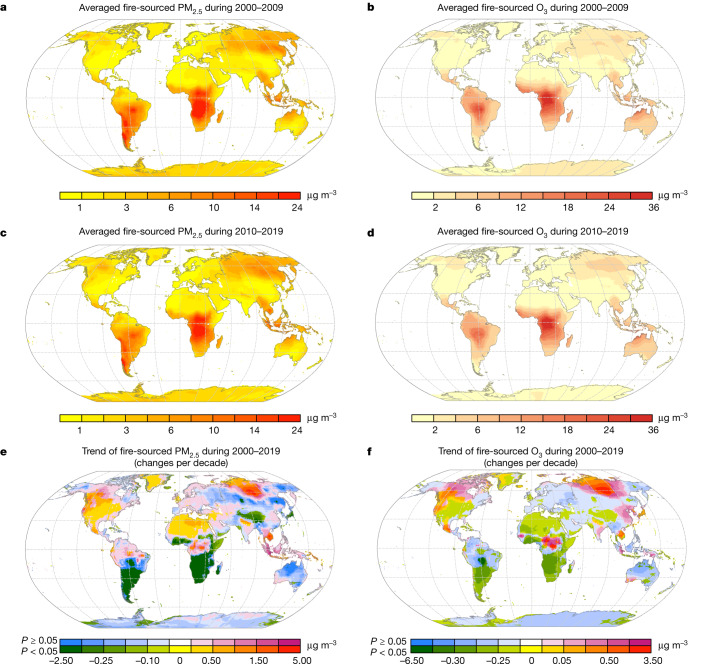
Global maps of estimated concentrations. **a**–**f**, Maps of LFS PM_2.5_ (**a**,**c**,**e**) and O_3_ (**b**,**d**,**f**) concentration in the first (**a**,**b**) and second (**c**,**d**) decades of 2000–2019, and the estimated trend (**e**,**f**) during the period. For each 0.25° × 0.25° grid, the trend from 2000 to 2019 was fitted using all annual concentrations during the period (not just 2000 and 2019 data) with a linear regression. *P* (**e**,**f**) indicates the *P* values for long-term trends, with *P* < 0.05 indicating a statistically significant trend. Source data

The global spatial distributions of fire-sourced PM_2.5_ and O_3_ were generally similar in 2000–2009 and 2010–2019 (Fig. 1), with Central Africa exposed to the highest levels of wildfire PM_2.5_ and O_3_, followed by Southeast Asia, South America and North Asia (Siberia). There were also some other regional hotspots, including north-western Australia, and western USA and Canada. From 2000 to 2019, fire-sourced PM_2.5_ showed statistically significant increasing trends in central and northern Africa, North America, Southeast Asia, Amazon areas in South America, Siberia and northern India, whereas notable decreasing trends were found in southern parts of Africa and South America, northwest China and Japan. Fire-sourced O_3_ also showed similar statistically significant increasing trends in Central Africa, Siberia, western USA and Canada, Mexico, Southeast Asia and northern India, and similar decreasing trends in northwest China and southern parts of Africa and South America; however, its trends in Amazon areas, central and eastern USA, Northern Africa, Japan and Indonesia were in the opposite direction of the trends of fire-sourced PM_2.5_ in those areas.

**Fig. 2:**
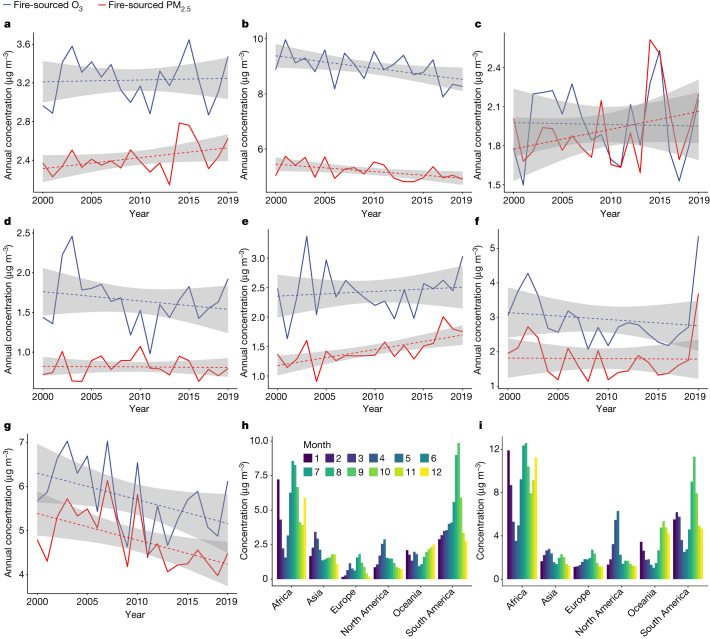
Global and continent-specific trends and seasonal patterns. **a**–**i**, The long-term trend (global (**a**), Africa (**b**), Asia (**c**), Europe (**d**), North America (**e**), Oceania (**f**) and South America (**g**)) and seasonal pattern of population-weighted average fire-sourced PM_2.5_ (**h**) and O_3_ (**i**) from 2000 to 2019 for the globe and six continents. The dashed lines in **a**–**g** denote point-estimates of fitted trend by linear regression and the shaded areas denote the corresponding 95% confidence intervals. Source data

The population-weighted average fire-sourced PM_2.5_ and O_3_ across the globe and six continents fluctuated substantially over the 2000–2019 period (Fig. 2), with different trends and seasonal patterns observed on different continents. The peak months of fire-sourced PM_2.5_ and O_3_ were June to September and December to January for Africa, March to April for Asia, July to August for Europe, April to May for North America, November to January for Oceania and August to October for South America.

Globally, the annual population-weighted average fire-sourced PM_2.5_ and O_3_ were 2.5 µg m^−3^ and 3.2 µg m^−3^ in 2010–2019, accounting for 6.1% and 3.6% of all-source PM_2.5_ and O_3_, respectively (Extended Data Table 1a). The annual population-weighted average wildfire PM_2.5_ from 2000 to 2019 showed increasing trends over the globe (0.11 µg m^−3^ increase per decade, *P* = 0.072 for trend) and in North America (0.27 µg m^−3^ increase per decade, *P* = 0.001 for trend), but decreasing trends in Africa (−0.27 µg m^−3^ per decade, *P* = 0.020 for trend) and South America (−0.61 µg m^−3^ per decade, *P* = 0.012 for trend). The annual population-weighted average wildfire O_3_ also showed decreasing trends in Africa (−0.45 µg m^−3^ per decade, *P* = 0.043 for trend) and South America (0.60 µg m^−3^ per decade, *P* = 0.012 for trend), but the trend was not significant for the globe or other continents (all *P* > 0.37 for trend).

**Extended Data Fig. 1:**
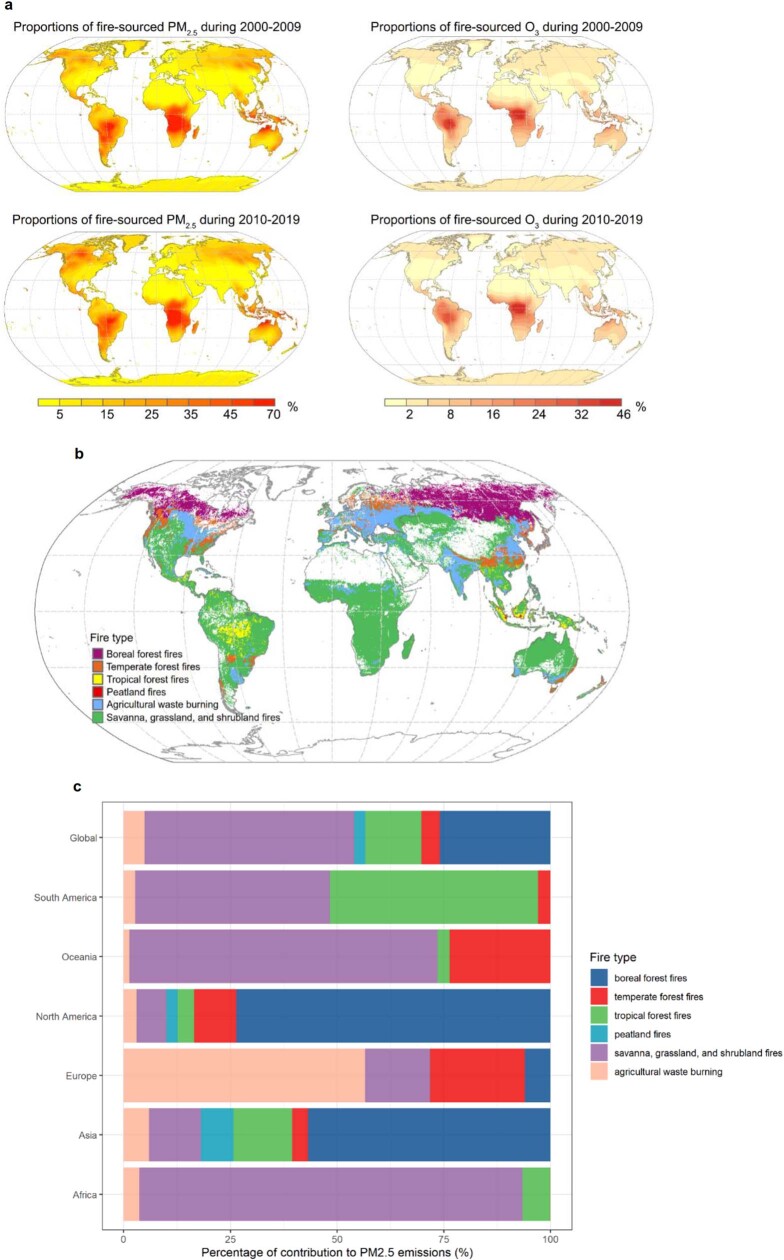
Landscape fires’ relative contributions to air pollution and the global distribution and relative contributions of different fire types. **a**, Maps showing the proportions of fire-sourced PM_2.5_ and O_3_ (among all-source PM_2.5_ and O_3_, respectively) during 2000–2009 and 2010–2019 across the globe. **b**, Map showing the dominant landscape fire type burned during 2000–2019 at 0.25° × 0.25° spatial resolution across the globe according to Global Fire Emissions Database (version 4.1 with small fires, GFED4.1s). The dominant fire type in each grid was determined based on highest proportion of dry matter burned for this grid during the period 2000–2019. Those white grids were grids without any landscape fire burned during the period. **c**, Stacked bar chart showing the relative contributions of different fire types to the fire-emitted PM_2.5_ across the globe and in each continent according to GFED4.1s. Here we only calculated the relative contribution to primary PM_2.5_ emitted from fires, without considering the secondary PM_2.5_ generated from fire emissions.

The proportions of fire-sourced PM_2.5_ and O_3_ among all sources showed similar spatial distributions for 2000–2009 and 2010–2019 (Extended Data Fig. 1a). The highest landscape fire contribution to PM_2.5_ was observed in Central Africa (up to 70%), followed by South America (approximately 40%), northern Australia (approximately 40%), Southeast Asia (approximately 30%), western USA and Canada (approximately 20% in 2000–2009, increased to approximately 30% in 2010–2019) and Northeast Asia (approximately 20%). The highest landscape fire contribution to O_3_ was also observed in Central Africa (up to 46%), followed by South America (approximately 30%), northern Australia (up to 20%) and Southeast Asia (up to 20%).

## Socioeconomic disparities in concentrations

**Fig. 3:**
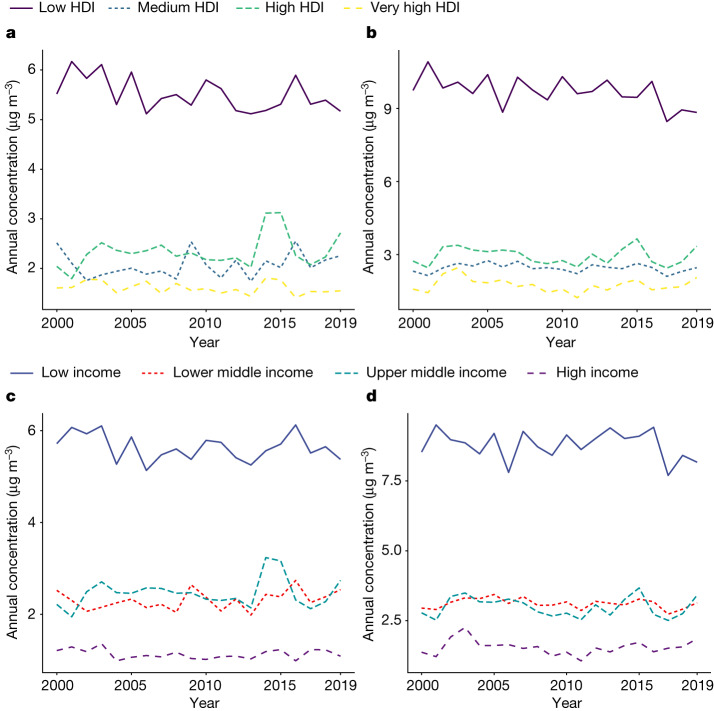
Socioeconomic disparities in exposure between countries. **a**–**d**, Annual population-weighted average fire-sourced PM_2.5_ (**a**,**c**) and O_3_ (**b**,**d**) from 2000 to 2019 by country HDI score (**a**,**b**) and income level (**c**,**d**). Source data

There were consistent socioeconomic disparities in the annual average fire-sourced PM_2.5_ and O_3_ concentrations (Fig. 3 and Extended Data Table 1a). Countries with a low Human Development Index (HDI) score and low income had the greatest exposure to fire-sourced air pollution, whereas countries with a very high HDI score and high income had the least exposure. The annual population-weighted average fire-sourced PM_2.5_ concentrations in countries with low HDI scores were 2.9- to 4.2-fold (varied in different years) those of countries with very high HDI scores during the period 2000–2019. These ratios for annual fire-sourced O_3_ (low HDI score versus very high HDI score) were 4.1 to 7.8. Similarly, annual fire-sourced PM_2.5_ and O_3_ concentrations in low-income countries were 4.5- to 6.2-fold, and 3.9- to 8.1-fold, respectively, those in high-income countries.

## Global population exposure to SFAP

We defined a substantial fire-sourced air pollution (SFAP) day as at least one of the following scenarios: (1) the daily average PM_2.5_ (all-source PM_2.5_) exceeded the 2021 daily guideline value (15 µg m^−3^) of the World Health Organization (WHO), and fire-sourced PM_2.5_ accounted for at least 50% of the daily PM_2.5_; (2) the daily maximum 8 h O_3_ (all-source O_3_) exceeded the WHO’s 2021 daily guideline value (100 µg m^−3^), and fire-sourced O_3_ accounted for at least 50% of the daily O_3_. The population exposures to SFAP were represented by three metrics, comprising annual total person-days, annual average days per person and annual total number of people exposed to SFAP. One person-day refers to one person exposed to 1 day of the SFAP; thus the total exposed person-days can be viewed as the total population exposure level to SFAP.

**Extended Data Fig. 2:**
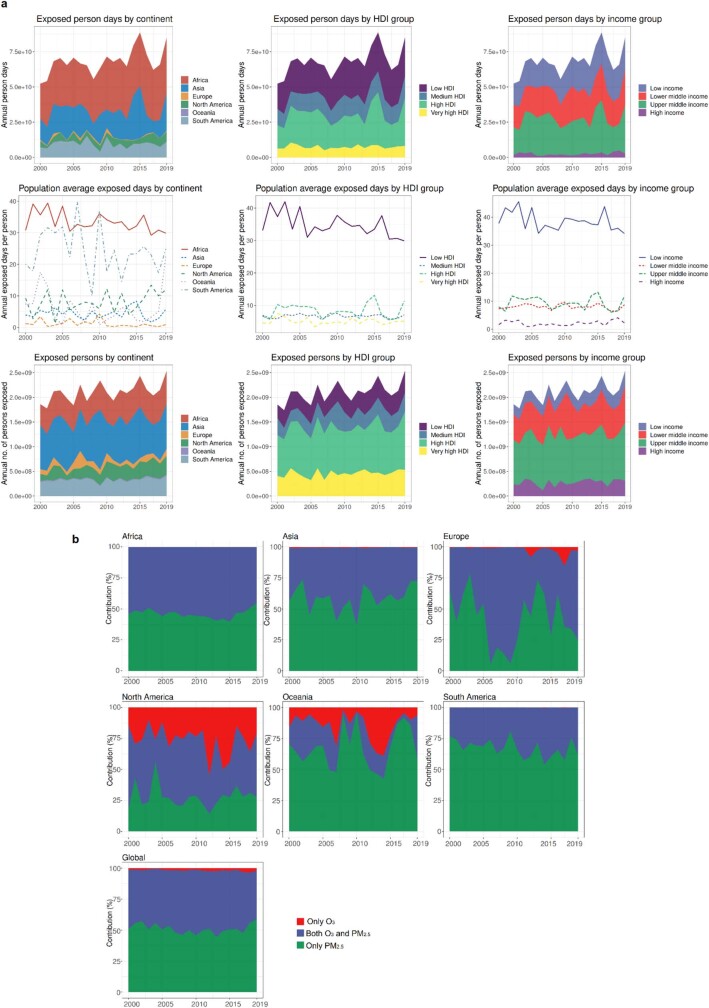
Annual exposures for main subgroups and the relative contribution of fire-sourced PM_2.5_ and O_3_ to the exposure. **a**, Stacked area charts and line plots showing the annual population exposure to substantial fire-sourced air pollution (SFAP) from 2000 to 2019 by continent, countries’ human development index (HDI) and income level. **b**, Stacked area charts showing the relative contribution of fire-sourced PM_2.5_ and O_3_ to the person days exposed to substantial fire-sourced pollution (SFAP) from 2000 to 2019 by continent.

The global total number of exposed person-days increased significantly from 63.2 billion per year during the period 2000–2009 to 72.8 billion per year during 2010–2019 (*P*  = 0.010 for trend, an increase of 8.6 billion person-days per decade) (Extended Data Table 1b and Extended Data Fig. 2a). This increase was mainly due to population growth, as the average exposed days per person per year increased only slightly from 9.7 days during 2000–2009 to 9.9 days during 2010–2019. In each year during 2000–2009, 2.04 billion people, on average, were exposed to at least 1 day of SFAP across the globe, and this number rose to 2.18 billion people per year during 2010–2019 (*P* = 0.007 for trend, a 190.1 million-person increase per decade).

There were notable disparities in the population exposures to SFAP between different continents. Africa experienced the largest proportion of exposed person-days (approximately 50% of global total) over the period 2000–2019, followed by Asia (more than 25%) (Extended Data Table 1b and Extended Data Fig. 2a). Africa experienced the fastest increase in exposed person-days (an increase of 6.0 billion person-days per decade, *P* < 0.001 for trend) from 2000 to 2019. North America also saw a significant increasing trend (an increase of 1.5 billion person-days per decade, *P*  = 0.042 for trend).

Africa had the highest average number of days exposed to SFAP per person per year (32.5 days per person per year during 2010–2019), despite a significant decrease (−2.5 days per decade, *P* = 0.029 for trend) since 2000–2009. South America had the second highest average number of exposed days (23.1 days per person per year during 2010–2019), whereas other continents were generally exposed to less than 10 days per person per year, except for a few outliers (for example, 23 days in 2019 for Oceania), and Europe had the lowest average number of exposed days (approximately 1 day per person per year) (Extended Data Table 1b and Extended Data Fig. 2a).

Asia had the largest annual population size exposed to at least 1 day of SFAP (803.1 million people per year during the period 2000–2019, 36.8% of the global total), followed by Africa (596.4 million, 27.4%), South America (342.5 million, 15.7%) and North America (319.2 million, 14.7%) (Extended Data Table 1b and Extended Data Fig. 2a). The fastest increase in exposed population size was seen in North America (a 109.1 million-person increase per decade, *P* = 0.001 for trend), then Africa (an 83.5 million-person increase per decade, *P* < 0.001 for trend) and South America (a 30.4 million-person increase per decade, *P* = 0.096 for trend).

Most of the person-days exposed to SFAP were characterized by substantial fire-sourced PM_2.5_ pollution only (approximately 50% globally) and substantial fire-sourced PM_2.5_ and O_3_ simultaneously (approximately 45% globally). Fire-sourced PM_2.5_ contributed to SFAP much more than fire-sourced O_3_ in all continents except North America and Oceania, where around or more than 25% of total exposed person-days were due to substantial fire-sourced O_3_ only in some years (Extended Data Fig. 2b).

## Socioeconomic disparities in SFAP exposure

Overall, low- and middle-income countries shared more than 96% of global total exposed person-days and over 86% of global total exposed people (Extended Data Table 1b, Extended Data Fig. 2a). The annual average number of days exposed to SFAP was three times greater for countries with a low HDI score and low income (30–45 days per person per year) than for countries with other HDI scores and income groups (generally <10 days per person per year).

Despite a decreasing trend of the annual exposed days per person, the countries with low HDI scores saw the largest increasing trends for both exposed person-days and exposed people (*P* < 0.001 for trends), whereas the countries with very high HDI scores had the smallest increasing trends in these two metrics (Extended Data Table 1b). This pattern was similar when comparing different income groups.

## Leading countries in exposure

All leading countries (top ten) for five different exposure metrics were low- and middle-income countries, except for the USA, Japan and Chile.

**Fig. 4:**
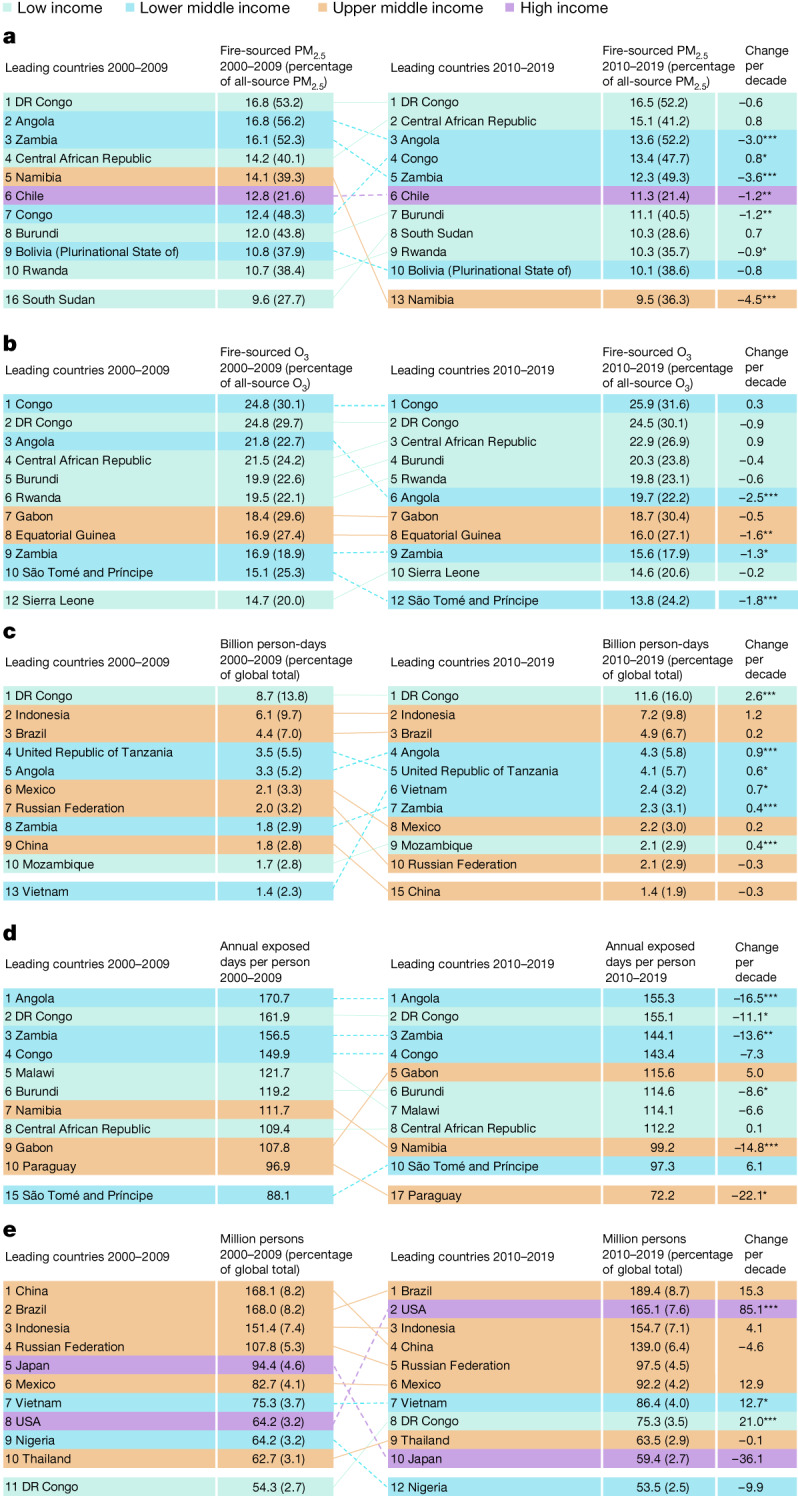
Leading countries with greatest exposures. **a**–**e**, Top ten countries with greatest annual population exposure levels to fire-sourced air pollution in 2000–2009 and 2010–2019, using five different exposure metrics: annual population-weighted average fire-sourced PM_2.5_ concentration (µg m^–3^) (**a**); annual population-weighted average fire-sourced O_3_ concentration (µg m^–3^) (**b**); annual person-days exposed to SFAP (**c**); annual population average number of days exposed to SFAP (**d**); and annual total persons exposed to at least one day of SFAP (**e**). *P < 0.05 for long-term trend; **P < 0.01; ***P < 0.001. Source data

In 2010–2019, the top five countries in population-weighted average fire-sourced PM_2.5_ concentrations were the Democratic Republic of the Congo (DR Congo), the Central African Republic, Angola, Congo and Zambia (all greater than 12 µg m^−3^); the top five countries in population-weighted average fire-sourced O_3_ concentrations were Congo, DR Congo, the Central African Republic, Burundi and Rwanda (all greater than 23 µg m^−3^) (Fig. 4a,b). The list of countries with the highest annual average number of days exposed to SFAP per person was similar, with Angola, DR Congo, Zambia, Congo and Gabon as the top five countries (all greater than 115 days per year during the period 2010–2019) (Fig. 4d). All top ten countries in these three exposure metrics were sub-Saharan African countries (mostly Central African countries), with three exceptions (Chile, Bolivia and Paraguay, in South America).

By contrast, the leading countries in total person-days and people exposed to SFAP were more dominated by several populous countries (Fig. 4c,e). In 2010–2019, the top five countries in terms of total exposed person-days were DR Congo (11.6 billion person-days per year), Indonesia (7.2 billion), Brazil (4.9 billion), Angola (4.3 billion) and Tanzania (4.1 billion); the top five countries in terms of total exposed people were Brazil (189.4 million people per year), the USA (165.1 million), Indonesia (154.7 million people), China (139.0 million) and the Russian Federation (97.5 million). DR Congo had consistently been the country with the largest total exposed person-days in 2000–2009 and 2010–2019, and it showed notable increasing trends in both exposed person-days (an increase of 2.6 billion person-days per decade, *P* < 0.001 for trend) and exposed people (a 21.0 million-person increase per decade, *P* < 0.001 for trend).

The rankings of these exposure metrics changed over time. A notable change was the USA, which ranked only eighth in the total number of exposed people in 2000–2009, but rose to second in 2010–2019 (an 85.1 million-person increase per decade, *P* < 0.001 for trend).

## Discussion

Through a validated machine learning approach with inputs from chemical transport models, ground-based monitoring stations and gridded weather data^7,8,26^, we estimated and mapped the global daily LFS PM_2.5_ and O_3_ at a 0.25° × 0.25° spatial resolution between 2000 and 2019. This filled a critical data gap, particularly for areas without monitoring stations. With these data and high-resolution global population distribution data, we made by far the most comprehensive assessment of global population exposure to LFS air pollution in the world, to the best of our knowledge.

Our assessment highlighted the severity and scale of the fire-sourced air pollution and a notable increasing trend in the population exposure. Short-term exposure to fire-sourced air pollution has many adverse health impacts, including increased mortality and exacerbations of cardiorespiratory conditions^6,7,27^. The large quantity and increasing trend of the population exposure to SFAP suggests that landscape fire air pollution is an increasing public health concern. Addressing this concern needs multisectoral efforts to reduce landscape fires and prevent adverse health impacts of landscape fire air pollution. Landscape fires can be partially reduced through effective evidence-based fire management, as well as appropriate planning and design of natural and urban landscapes^4^. Policy change may help to reduce some landscape fires caused directly by humans, such as agricultural waste burning in Europe, India, eastern China and the USA (Extended Data Fig. 1b), and the fires deliberately set by humans to convert wildlands to agricultural or commercial lands (common in South America and South and Southeast Asia^24,28^).

However, unplanned wildfires are more difficult to control, as evidenced by the fact that aggressive fire suppression actually contributed to the extreme wildfires in western USA in recent decades because of fuel accumulation^29^. Wildfires are also an essential component of Earth’s ecosystem and cannot be totally prevented^4^. Therefore, a considerable proportion of human exposure to LFS air pollution seems to be unavoidable. This highlights the importance of health protection measures against exposure. Unfortunately, existing measures that individuals can take to protect themselves from landscape air pollution, such as relocation, staying indoors, using air purifiers with effective filters and wearing N95 or P100 face masks, all have limitations and are not feasible for people with limited resources^6^; thus it is urgent to develop more cost-effective health protection measures.

The observed increasing global trend of fire-sourced PM_2.5_, although only marginally significant, seems to be inconsistent with the previously reported decline in global burned areas in previous decades^30,31^. However, the decreased global burned areas were mainly in savannas and grasslands because of cropland and pasture expansion, whereas burned areas in forests increased^30,31^. Forests provide much more fuels per unit of burned area than savannas and grasslands^31^, and also have a much larger quantity of emissions per unit of dry biomass burned^32^. Therefore, the increased PM_2.5_ emissions from forest fires tends to exceed the decline in PM_2.5_ emissions from savannas and grassland fires. This could explain our observed increasing trend of global fire-sourced PM_2.5_ despite the decline in global burned areas.

It was expected that the temporal trend of fire-sourced O_3_ was not perfectly consistent with the trend of fire-sourced PM_2.5_. Ground-level or tropospheric O_3_ is a secondary pollutant generated from photochemical reactions between volatile organic compounds (VOCs) and nitrogen oxides (NO_*x*_) under sunlight^33,34^. The generation of fire-sourced O_3_ can thus be affected by many non-fire factors, such as VOCs and NO_*x*_ from industrial and traffic sources, and weather conditions (for example, reduced sunlight during smoky days)^33,34^. In particular, the impacts of VOCs and NO_*x*_ emissions on O_3_ formation are nonlinear^34^; thus whether the NO_*x*_ and VOCs emitted from landscape fires can increase the ground-level O_3_ level is often uncertain. This uncertainty was supported by our results showing that the estimated fire-sourced O_3_ could even decrease during wildfire periods, compared with pre-wildfire periods, in two out of the ten selected wildfire events (Extended Data Fig. 6b). The relatively uncertain impacts of fires on surface O_3_ could explain why the global fire-sourced O_3_ did not show a significant increasing trend like the wildfire PM_2.5_.

Our assessment highlighted the substantial geographical disparities in the population exposures to fire-sourced air pollution. There were several hotspots, including Central Africa, Southeast Asia, South America and Siberia, which experienced the most severe fire-sourced air pollution during the years 2000–2019. North America saw the most significant increases in fire-sourced PM_2.5_ concentrations and the population size exposed to SFAP. The geographical distributions of fire-sourced PM_2.5_ and O_3_ in our study were generally consistent with a previous map of global landscape fire density^35^, but were very different from the global map of meteorological fire danger, that is, the fire weather index (FWI)^36^. For example, the FWI value was very high in North Africa, but low in Central Africa and Siberia. This suggests that the FWI may not be able to capture the actual landscape fire density and the related air pollution, and thus should be used with caution in monitoring and managing landscape fire impacts.

Our assessment also highlighted the socioeconomic disparities in population exposures to fire-sourced air pollution. The disparity could be partly explained by the fact that many low- and middle-income countries are located in hot and dry areas that are prone to landscape fires^4^. The disparity could also be partly due to some other factors, such as that less industrialized countries have more agricultural waste burning and deliberate burning of forests for agricultural or other purposes, and poorer management or control of wildfires^4,37^. More studies are warranted to understand the underlying causes of the disparity, which will help to narrow the gaps. However, our finding does not mean that LFS air pollution is not serious or not important in high-income countries. In fact, we also identified regional hotspots of high levels of fire-sourced air pollution in Australia, the USA and Canada, which were caused by their catastrophic wildfire events in recent years^6^. The value of our study is in highlighting that many low- and middle-income countries have more serious fire-sourced air pollution than that of the high-income countries (for example, the USA, Australia, Canada and western and northern Europe) that attracted the most media and research attention. More attention is needed for those neglected countries to mitigate their fire-sourced air pollution and the related health consequences.

Because the increasing severity and frequency of wildfires are related to anthropogenic climate change^1–4^, our finding about the socioeconomic disparities provides further evidence of climate injustice, that is, those least responsible for climate change suffer the most from its consequences^38,39^. A vivid example in our study is the DR Congo, a low-income country with the world’s highest fire-sourced PM_2.5_ concentrations. Its anthropogenic carbon dioxide emission per capita was among the lowest in the world (0.03 tons versus the world average of 4.76 tons in 2019^40^). The global socioeconomic disparities in population exposure to fire-sourced air pollution are likely to lead to even larger disparities in health consequences related to the exposure, as poorer countries have more limited resources to protect health against this hazard. This exemplifies how climate change is exacerbating global health inequality. To address this climate injustice, more resources should be allocated to low- and middle-income countries to prevent the health risks from exposure to landscape fire air pollution.

Robust projections suggest that climate change will increase wildfire frequency and intensity in future^4,5,41–43^. Therefore, global fire-sourced air pollution is likely to continue to be an increasingly important public health concern in the next decades. Immediate actions to limit the magnitude of climate change are needed. A projection suggests that wildfire frequency will substantially increase across 74% of the global lands by 2100 under a scenario of high greenhouse gas emissions^43^. However, if the global mean temperature increase could be limited to 2.0 °C or 1.5 °C above pre-industrial levels, over 60% or 80%, respectively, of the increase in wildfire exposure could be avoided^43^. The 1.5 °C target remains reachable, if the world can reduce annual carbon emissions by an extra 28 gigatons of carbon dioxide equivalent (approximately 50% of current emission levels) by 2030^44^.

The main strength of our study, compared with previous studies of population exposure assessment of landscape fires, is that we evaluated the population exposure to fire-sourced air pollution, rather than just direct exposure to the flames and heat of landscape fires^21,45^. Fire-sourced air pollution can often travel hundreds (sometimes even thousands) of kilometres and affect much larger populations, causing greater health consequences^4,6^. For example, previous data found that 260,000 people suffered from direct exposure to landscape fires in 2018^45^, but this number was only about 0.01% of the population (2.15 billion in 2018) exposed to SFAP. The other data source estimated the annual number of person-days exposed to landscape fires (direct exposure) for each country in the world. Consistent with our study to some extent, it found that DR Congo experienced the largest number of person-days of direct exposure to landscape fires (15,300 person-days per year during 2017–2020)^21^. Again, this number was only about 0.001% of this country’s person-days exposed to SFAP (12.0 billion in 2019).

Our study generated a database that can be used for evaluating and tracking the population exposure to LFS air pollution (both PM_2.5_ and O_3_) across the globe, which is superior to previous studies focusing on fire-related PM_2.5_ in specific regions (the USA^9–18^, Europe^19,20^ and Brazil^7^). Our estimated fire-sourced PM_2.5_ showed a high level of agreement (Pearson correlation coefficient *r* = 0.88) with the estimated smoke PM_2.5_ by Childs et al.^17^. The high level of agreement with Childs et al.^17^ is supported by another study, which found that the summer wildfire smoke PM_2.5_ estimated by the satellite-based smoke plume approach and the GEOS-Chem approach showed generally similar spatial and temporal distribution in the USA over the period 2006–2016^18^. However, the smoke PM_2.5_ estimates of Childs et al.^17^ covered only the contiguous USA because it relied on a satellite-based smoke plume polygon product (available only in the contiguous USA and Alaska^46^) to define days and locations covered by landscape fire smoke^17^. The smoke PM_2.5_ tends to be a conservative measure of fire-sourced PM_2.5_ because of the limitations of the satellite-based smoke polygon product, for example, undetected plumes during night time and under cloud cover and in the scenarios when the smoke is dilute and difficult to detect^17^. GEOS-Chem also has some limitations, as we discuss later, so it is still not conclusive which approach is better in terms of accuracy, but the GEOS-Chem approach definitely has the advantage of global coverage.

Two previous studies also used chemical transport model simulations to assess global exposure to fire-sourced PM_2.5_ during 1997–2006 and 2016–2019^22,23^. These two studies observed global spatial distribution patterns of fire-sourced PM_2.5_ that were similar to those observed by us, but our study has the advantage of further calibrating chemical transport model outputs against air quality stations with a machine learning approach. According to our spatial CV and validation against the smoke PM_2.5_ by Childs et al.^17^, the calibration approach substantially improved the accuracy of the estimated all-source PM_2.5_ and O_3_, as well as fire-sourced PM_2.5_ (Extended Data Figs. 4 and 7). With this approach, we also estimated the world’s first daily fire-sourced O_3_ data with global coverage. Moreover, we have a much longer study period and have conducted more comprehensive analysis of the population exposure levels using various metrics for both fire-sourced PM_2.5_ and O_3_, at several spatial–temporal levels (global/regional/national, yearly/monthly/daily). Overall, our study provides the most accurate and comprehensive data at present for policymakers and the public to manage and mitigate LFS air pollution at global scale. The generated database also forms a critical basis for many future applications, such as evaluating various health impacts of this environmental hazard^6^, and estimating corresponding attributable mortality, morbidity and health-care costs^19,22^.

Several limitations of our study should be acknowledged. PM_2.5_, O_3_ and carbon monoxide (CO) are the main pollutants of public health concern during wildfire events^47^, but we did not quantify CO from landscape fires because of data unavailability. Previous studies suggest that the impacts of wildfires on CO are generally confined to the immediate fire areas^9,48^, which can be explained by the photochemical loss of CO (that is, photochemical oxidation of CO and hydrocarbons in the presence of nitrogen oxides produces O_3_) during long-distance transport of biomass plumes^49^. Therefore, the unavailability of CO would be expected to have minimal impact on the estimation of the population exposure to fire-related air pollution. Other limitations, including the uncertainties of the fire emission inventory, the GEOS-Chem simulations, and machine learning models, are discussed in detail in Methods.

In conclusion, we conducted a comprehensive assessment of global population exposure to LFS air pollution. We found that billions of people worldwide were exposed to substantial LFS air pollution, and the exposure levels were particularly high in several hotspots (Central Africa, Southeast Asia, South America and Siberia) and in the least developed countries.

## Methods

### Data collection

#### Monitoring station data

We collected global air quality monitoring station data from several sources. Monitoring data for the USA were downloaded from the US Environmental Protection Agency (US EPA)^50^. Data for China were downloaded from the China National Environmental Monitoring Centre (http://www.cnemc.cn/en/). Data for member countries of the European Economic Area were downloaded from the European Environment Agency^51^. Data for Australia were sourced from the National Air Pollution Monitoring Database, which integrated all available monitoring data from Australian state-specific governmental agencies^52,53^. Data for New Zealand were downloaded from Environment Canterbury (http://data.ecan.govt.nz/Catalogue/Method?MethodId=98). Data for Chile were downloaded from its National Air Quality Information System (https://sinca.mma.gob.cl/index.php/region/index/id/II). Data for South Africa were downloaded from the South African Air Quality Information System (https://saaqis.environment.gov.za/). Data for two African countries (Algeria and Nigeria) were downloaded from AirQo (https://www.airqo.net/, only PM_2.5_ data available).

Data for other countries and territories were downloaded from OpenAQ (https://openaq.org/). To ensure data quality, we used data from reference-grade monitoring stations only.

**Extended Data Fig. 3:**
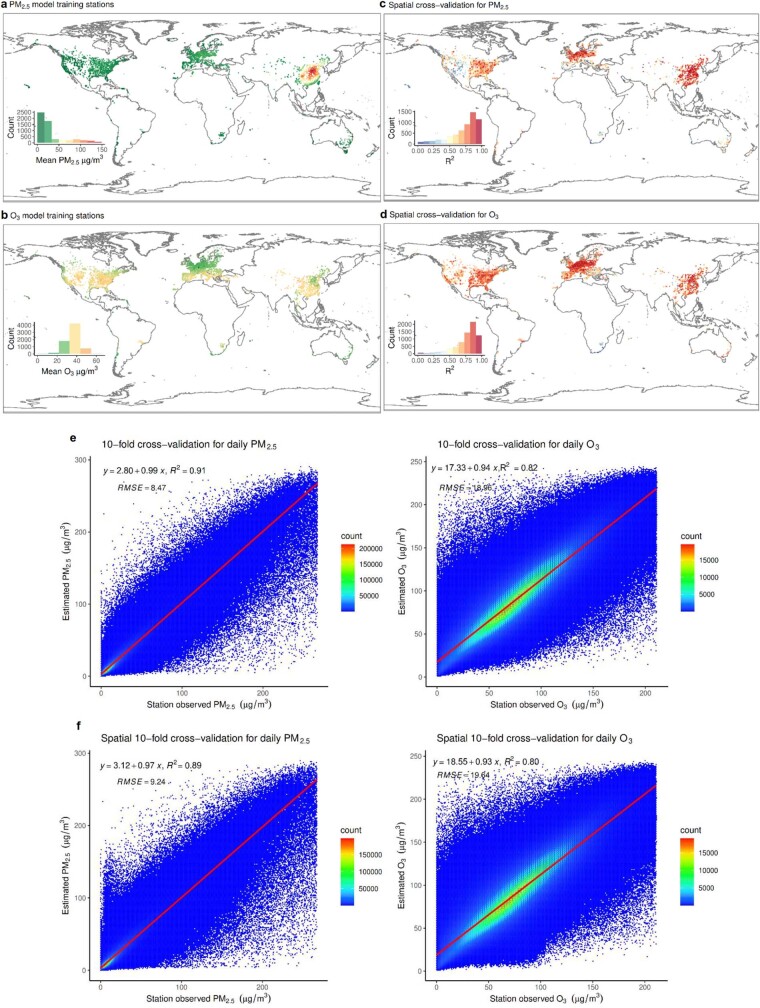
Locations of model training stations and model performance of cross-validations. **a** and **b**, Maps showing the geographical distribution of air quality monitoring stations of PM_2.5_ (**a**) and O_3_ (**b**) used for machine learning model training, the mean PM_2.5_ or O_3_ refers to the average value of all available valid observations for each station. **c** and **d**, Maps showing the station-specific R^2^ in the spatial 10-fold cross-validation of the random forest models for estimating all-source daily average PM_2.5_ (**c**) and daily maximum 8-hour average O_3_ (**d**). **e**, Density scatter plots showing the overall performance of the machine learning models for estimating all-source daily average PM_2.5_ (left) and daily maximum 8-hour average O_3_ (right) based on general 10-fold cross-validation. RMSE, root mean squared error. **f**, Density scatter plots showing the overall performance of the machine learning models for estimating all-source daily average PM_2.5_ (left) and daily maximum 8-hour average O_3_ (right) based on spatial 10-fold cross-validation.

After a data cleaning and quality control process (Supplementary Information), we kept 9,528,179 valid daily average PM_2.5_ observations of 5,661 stations from 73 countries and territories and 21,097,834 valid daily 8 h maximum O_3_ observations of 6,851 stations from 58 countries and territories (Supplementary Tables 1 and 2 and Extended Data Fig. 3). Both PM_2.5_ and O_3_ station data covered the whole period between 2000 and 2019, although the data period varied by country and stations. We unified all units of PM_2.5_ and O_3_ as µg m^−3^, consistent with the latest WHO air quality guidelines 2021^54^. For O_3_, 1 part per billion (ppb) was approximated as 1.96 µg m^−3^, assuming a standard air pressure and temperature (25.5 °C and 101.325 kPa)^55^.

#### Chemical transport model simulations

As described previously^7,8,26^, we used the three-dimensional chemical transport model GEOS-Chem (v.12.0.0) based on O_3_–NO_*x*_–hydrocarbon–aerosol chemical mechanisms to estimate daily total (that is, all-source) and fire-sourced PM_2.5_ and O_3_ concentrations at 2.0° latitude by 2.5° longitude horizontal resolution (about 220 km × 280 km) during years 2000–2019 across the globe. Daily fire-sourced PM_2.5_ and O_3_ concentrations were estimated as the differences between GEOS-Chem simulations with and those without fire emissions. The fire emission data came from the Global Fire Emissions Database (v.4.1 with small fires, GFED4.1s)^56^, which captured aerosol emissions from six fire sources (boreal forest fires; tropical forest fires; savanna, grassland and shrubland fires; temperate forest fires; peatland fires; and agricultural waste burning) according to satellite retrieval of burned areas and active fire information^26^. On the basis of the GFED4.1s data, the relative contributions of different fire types to the fire-emitted PM_2.5_ varied by continent (for example, North America and Asia are characterized by high proportions of boreal forest fires; Oceania and Africa by savanna, grassland and shrubland fires; South America by tropical forest fires; and Europe by agricultural fires) (Extended Data Fig. 1c). We also provide the dominant landscape fire type burned during the period 2000–2019 at 0.25° × 0.25° spatial resolution across the globe in Extended Data Fig. 1b, which suggests that the peatland fires burned mainly in Southeast Asia.

#### Meteorological data

We derived hourly meteorological data at 0.25° × 0.25° spatial resolution from the fifth-generation European Centre for Medium-Range Weather Forecasts Reanalysis (ERA5)^57^. ERA5 combines model results with worldwide weather observations into a globally complete and consistent dataset using the laws of physics. Hourly records were used to calculate daily metrological parameters according to the local time zone of each grid. These daily metrological parameters included daily mean/minimum/maximum 2 m (that is, at 2 m above the surface of the earth) ambient temperature (*T*_mean_, *T*_min_ and *T*_max_, all calculated from 24-hourly records of 2 m ambient temperature), daily temperature variability (TV, standard deviations of 24-hourly 2 m temperatures), daily mean 2 m dew point temperature (*T*_dew_mean_), daily mean eastward component of 10 m wind (Wind_u, 10 m refers to 10 m above the surface of the earth), daily mean northward component of 10 m wind (Wind_v), daily total precipitation (Precip), daily mean surface air pressure (Pressure) and daily mean downward ultraviolet radiation at the surface (UV). Daily mean relative humidity (RH) was calculated from *T*_mean_ and *T*_dew_mean_ using the humidity R package^58^.

#### Population data

We collected annual population count data at 30 arcseconds (about 1 km^2^) spatial resolution across the globe during the years 2000–2019 from the WorldPop project^59^. Specifically, we downloaded the unconstrained global mosaics data (approximately 1 km × 1 km spatial resolution). This dataset was generated using the top-down unconstrained approach to disaggregate administrative unit-based census and projection counts for each year to grid cell-based population counts, by using a set of detailed geospatial predictors and a random forest machine learning model^60^. We aggregated the gridded population counts to 0.25° × 0.25° spatial resolution to match the air pollution data. For each country or territory in each year, all grid-specific population counts within its boundary were further multiplied by an adjustment coefficient (that is, the population size of that country or territory reported by the United Nations/sum of all grid-specific population counts within the boundary). This adjustment ensured that the country-specific population counts were consistent with data from the United Nations^61^.

#### Socioeconomic data

Countries were classified as low-income countries (gross national income (GNI) per capita ≤ US$1,035), lower-middle-income countries (US$1,035 < GNI per capita ≤ US$4,045), upper-middle-income countries (US$4,045 < GNI per capita ≤ US$12,535) and high-income countries (GNI per capita > US$12,535) according to the World Bank’s 2019 criteria^62^. Country-level HDI data in 2019 were downloaded from the United Nations Development Programme (UNDP). HDI is a unified measure of average achievement in key dimensions of human development, including a long and healthy life, being knowledgeable (educated) and having a decent standard of living. HDI scores range from 0 to 1, and can be divided into four tiers: very high (0.8 to 1.0), high (0.70 to 0.79), medium (0.55 to 0.69) and low (less than 0.55)^63^.

### Estimating fire-sourced PM_2.5_ and O_3_

We estimated global fire-sourced PM_2.5_ and O_3_ at 0.25° × 0.25° spatial resolution with three steps. In step one, we downscaled daily total and fire-sourced PM_2.5_ and O_3_ derived from GEOS-Chem to 0.25° × 0.25° spatial resolution using the inverse distance weighted spatial interpolation^8,64^.

In step two, downscaled GEOS-Chem outputs were further calibrated to match ground monitoring station observations based on a random forest machine learning algorithm. Briefly, the downscaled GEOS-Chem outputs and gridded meteorological data were linked to ground monitoring stations based on longitude and latitude, which generated the model training datasets. Then we trained two random forest models to predict station-observed total PM_2.5_ (PM_2.5_station_) and O_3_ (O_3_station_) separately, with the following equations:

1 $$\begin{array}{l}{{\rm{PM}}}_{2.5\_{\rm{station}}}\,=\,f({{\rm{PM}}}_{2.5\_{\rm{chem}}\_{\rm{total}}}{,T}_{{\rm{mean}}}{,T}_{\max }{,T}_{\min },{\rm{TV}},{\rm{RH}},\\ \,\,\,{\rm{Wind}}\_{\rm{u}},{\rm{Wind}}\_{\rm{v}},{\rm{Precip}},{\rm{Pressure}},{\rm{UV}},{\rm{Year}},\\ \,\,\,{\rm{Month}},{\rm{DOW}},{\rm{DOY}},{\rm{Lon}},{\rm{Lat}})\end{array}$$

2 $$\begin{array}{l}{{\rm{O}}}_{3\_{\rm{station}}}\,=\,f({{\rm{O}}}_{3\_{\rm{chem}}\_{\rm{total}}}{,T}_{{\rm{mean}}}{,T}_{\max }{,T}_{\min },{\rm{TV}},{\rm{RH}},{\rm{Wind}}\_{\rm{u}},\\ \,\,{\rm{Wind}}\_{\rm{v}},{\rm{Precip}},{\rm{Pressure}},{\rm{UV}},{\rm{Year}},{\rm{Month}},{\rm{DOW}},\\ \,\,{\rm{DOY}},{\rm{Lon}},{\rm{Lat}})\end{array}$$

PM_2.5_chem_total_ and O_3_chem_total_ were downscaled daily total (all-source) PM_2.5_ and O_3_ derived from GEOS-Chem. *T*_mean_ to UV were ERA5 meteorological variables, as mentioned above. DOW was day of week (Monday to Sunday). DOY was day of year (1 to 366). Lon and Lat were longitude and latitude, respectively. *f* referred to the random forest algorithm fitted with the ranger R package^65^.

In step three, the daily total (all-source) PM_2.5_ (PM_2.5_est_total_) and O_3_ (O_3_est_total_) for each 0.25° × 0.25° grid (regardless of whether close to or far away from the training stations) across global lands were estimated using the trained random forest models (that is, machine learning calibration or bias correction algorithms found where training stations existed) and global seamless predictor data. Then the final estimated fire-sourced PM_2.5_ (PM_2.5_est_fire_) and O_3_ (O_2.5_est_fire_) were calculated as follows^7,8,64^:

3 $${{\rm{PM}}}_{2.5\_{\rm{est}}\_{\rm{fire}}}\,=\,{{\rm{PM}}}_{2.5\_{\rm{est}}\_{\rm{total}}}\times ({{\rm{PM}}}_{2.5\_{\rm{chem}}\_{\rm{fire}}}{/{\rm{PM}}}_{2.5\_{\rm{chem}}\_{\rm{total}}})$$

4 $${{\rm{O}}}_{2.5\_{\rm{est}}\_{\rm{fire}}}\,=\,{{\rm{O}}}_{3\_{\rm{est}}\_{\rm{total}}}\times ({{\rm{O}}}_{3\_{\rm{chem}}\_{\rm{fire}}}{/{\rm{O}}}_{3\_{\rm{chem}}\_{\rm{total}}})$$

The PM_2.5chem_fire_ and O_3_chem_fire_ refer to the downscaled fire-sourced PM_2.5_ and O_3_ from GEOS-Chem.

### Model performance evaluation

We used tenfold CV to test the performance of the random forest models and to find the optimal model parameters. Specifically, the whole model training dataset was randomly divided into ten approximately equal subsets. Each subset was then treated as a validation set to test the performance of the model trained in the remaining nine subsets (this was repeated ten times)^66^. We also used a spatial tenfold CV (that is, dividing all stations, rather than the dataset, into ten approximate equal subsets, then performing CV in a manner similar to that described above) to test the model’s prediction ability in new locations not in the training data (that is, spatial generalization ability of the model).

We tested the spatial generalization ability of the models further using a spatial cluster-based CV approach. Specifically, we conducted a *k*-means cluster analysis^67^ based on the Euclidean distances between stations based on their longitude and latitude, and the optimal number of spatial clusters was determined by selecting the minimum sum-of-squares distances within groups. As a result, we identified 75 spatial clusters for PM_2.5_ stations and 99 spatial clusters for O_3_ stations across the globe. We then used each cluster as a testing dataset and the remaining clusters as the training dataset to train and test our random forest model 75 and 99 times for PM_2.5_ and O_3_, respectively. Compared with spatial tenfold CV, in which the nearby stations could be allocated to training and testing datasets simultaneously, the spatial cluster-based CV increases the difficulty of the prediction task^68^ but is a more realistic test of the models’ prediction abilities in large remote areas with essentially no training stations (for example, many areas in Africa and South America; Extended Data Fig. 3a,b).

The model reached a high level of accuracy in estimating both daily average PM_2.5_ (tenfold CV, *R*^2^ = 0.91, RMSE = 8.47 µg m^−3^) and daily maximum 8 h O_3_ (tenfold CV, *R*^2^ = 0.82, RMSE = 18.96 µg m^−3^) (Extended Data Fig. 3e). The model also showed a similarly high level of accuracy in the spatial tenfold CV for both PM_2.5_ (*R*^2^ = 0.89, RMSE = 9.24 µg m^−3^) and O_3_ (*R*^2^ = 0.80, RMSE = 19.64 µg m^−3^) (Extended Data Fig. 3f), suggesting good spatial generalization abilities of the trained random forest models.

We calculated station-specific *R*^2^ based on the spatial tenfold CV. The median station-specific model performance among stations was comparable to overall model performance (median station-specific *R*^2^, 0.80 for PM_2.5_ and 0.72 for O_3_), with 90% of station-specific *R*^2^ values above 0.38 for PM_2.5_ and above 0.53 for O_3_. There were notable spatial variations of the station-specific model performance (Extended Data Fig. 3c,d). Although the model estimates showed a high level of agreement with station observations in most stations, a low level of agreement between model estimates and station observations was found in some PM_2.5_ stations in the middle and southwestern USA, Hawaiian islands, southern Europe, Africa, and western and inland Australia, and some O_3_ stations in Chile, South Africa and New Zealand.

**Extended Data Fig. 4:**
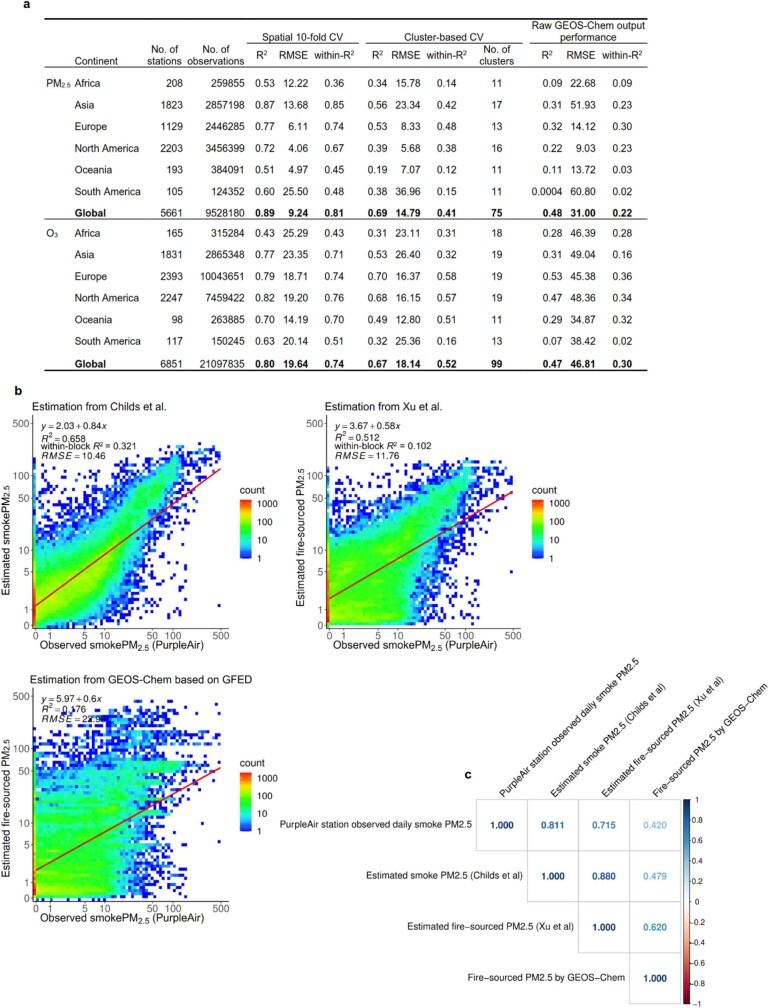
Overall cross-validation performance for estimating all-source PM_2.5_ and O_3_ and the accuracy of fire-sourced PM_2.5_ in validation against Childs et al. **a**, Table showing the global and continent-specific performance of the machine learning models estimating all-source daily PM_2.5_ and O_3_ based on spatial 10-fold and cluster-based cross-validation (CV), in comparison with the performance of the raw GEOS-Chem outputs. Some spatial cluster could be in two continents; this is why the global total cluster number is smaller than the sum of continent-specific cluster number. The unit of the root mean squared error (RMSE) is µg/m^3^ for both PM_2.5_ and O_3_. The continent-specific CV results was calculated by extracting each continent’s CV data from the global spatial 10-fold CV or global cluster-based CV. **b**, Density scatter plots showing the performance of our estimated fire-sourced PM_2.5_, GEOS-Chem simulated fire-sourced PM_2.5_, Childs et al’s estimated smoke PM_2.5_ in validation against smoke PM_2.5_ observed by PurpleAir stations. In this analysis, we included 68041 observations during 2016–2019 linked to 2147 PurpleAir stations in the contiguous US based on date, longitude and latitude. The smoke PM_2.5_ data and PurpleAir data were sourced from Childs et al.^17^. The within-block R^2^ could be interpreted as how much spatial variations of smoke PM_2.5_ within the 2.0° × 2.5° grid box can the model estimates (our estimated fire-sourced PM_2.5_ or Childs et al’s estimated smoke PM_2.5_) account for. **c**, Correlation matrix showing the Pearson correlations between our estimated fire-sourced PM_2.5_, GEOS-Chem simulated fire-sourced PM_2.5_, Childs et al’s estimated smoke PM_2.5_, and the smoke PM_2.5_ observed by PurpleAir station. The data used for panel c is the same as the data used for panel b.

We also estimated the within-*R*^2^ value of the spatial tenfold and cluster-based CV. The within-*R*^2^ value was calculated by regressing station observations on model estimates while controlling for the station and year fixed effects. As calculated by the *fixest* R package^69^, the within-*R*^2^ value of spatial tenfold CV was 0.81 and 0.74 for PM_2.5_ and O_3_, respectively (Extended Data Fig. 4a). This suggests that our random forest models can predict, on average, 81% and 74% of local temporal daily variations of all-source PM_2.5_ and O_3_, respectively, within a year, not just variations in average PM_2.5_ and O_3_ across locations and years.

As expected, the model performance of spatial cluster-based CV (PM_2.5_, *R*^2^ = 0.69, RMSE = 14.79 µg m^−3^; O_3_, *R*^2^ = 0.67, RMSE = 18.14 µg m^−3^) was lower than spatial tenfold CV, but still much higher than the performance of the raw GEOS-Chem outputs (PM_2.5_, *R*^2^ = 0.48, RMSE = 31.00 µg m^−3^; O_3_, *R*^2^ = 0.47, RMSE = 46.81 µg m^−3^) across the globe and in all continents. This suggests that our models can predict the daily all-source PM_2.5_ and O_3_ in large remote areas with no training data with an accuracy that is much higher than that of the raw GEOS-Chem outputs alone. Similarly, the within-*R*^2^ values for spatial cluster-based CV suggest that the model estimates can explain 41% and 52% of local temporal daily variations of all-source PM_2.5_ and O_3_, respectively, in spatial clusters not in the training data.

### Validation against smoke PM_2.5_

Childs et al.^17^ trained a machine learning model to predict the station-based smoke PM_2.5_ using meteorological factors, fire variables, aerosol measurements, and land use and elevation data, and the model was used to estimate daily smoke PM_2.5_ across the USA at 0.1° × 0.1° spatial resolution during the period 2006–2020. In their study, the station-based observed smoke PM_2.5_ was calculated through two steps: (1) days when smoke was overhead were defined as ‘smoke days’ based on satellite imagery-based plume classification (or simulated air trajectories originating at fires when clouds may be obscuring plumes), and days without smoke overhead were ‘non-smoke days’; (2) for each station on each smoke day, the observed smoke PM_2.5_ concentration was calculated as the station-observed all-source PM_2.5_ on the smoke day minus the background PM_2.5_, which was defined as the 3-year (previous year, current year and the next year) station- and month-specific median PM_2.5_ on non-smoke days. For example, if a smoke day was 10 January 2018 for station A, then its corresponding background PM_2.5_ was the median value of all daily PM_2.5_ observations of station A on non-smoke days in the January of each year during 2017–2019. The smoke PM_2.5_ on non-smoke days was assumed to be 0.

Because most of the training stations used by Childs et al.^17^ were also our training stations, directly validating our estimated fire-sourced PM_2.5_ against the observed smoke PM_2.5_ by the training stations of Childs et al.^17^ may have overfitting issues (that is, may overestimate our accuracy). To avoid this problem, we chose the observed smoke PM_2.5_ of PurpleAir stations (a kind of low-cost sensor) as our validation target. The PurpleAir stations were not included in our training stations nor in those of Childs et al.^17^, and thus could give a fair comparison of the accuracy of our estimated fire-sourced PM_2.5_ and the estimated smoke PM_2.5_ of Childs et al.^17^. The PurpleAir station data were collected and cleaned as detailed previously^17^, and its measured daily PM_2.5_ had been calibrated against US EPA reference-grade stations by Childs et al.^17^ before calculating its station-observed smoke PM_2.5_.

When validated against the PurpleAir station-observed smoke PM_2.5_, our estimates’ accuracy (*R*^2^ = 0.51, RMSE = 11.76 µg m^−3^) was lower than the accuracy of the estimates of Childs et al.^17^ (*R*^2^ = 0.66, RMSE = 10.46 µg m^−3^), but much higher than the accuracy of the fire-sourced PM_2.5_ from raw GEOS-Chem outputs (*R*^2^ = 0.18, RMSE = 22.96 µg m^−3^) (Extended Data Fig. 4b). Our estimated fire-sourced PM_2.5_ values were highly correlated with the estimated smoke PM_2.5_ by Childs et al.^17^ (Pearson correlation coefficient *r* = 0.88) (Extended Data Fig. 4c).

We also calculated with-block *R*^2^ by regressing station observations on model estimates while controlling for the block (that is, the 2.0° × 2.5° grid box of GEOS-Chem simulations) and date fixed effects. We found that our estimated fire-sourced PM_2.5_ could account for 10% (within-block *R*^2^ = 0.10) of the spatial variations of the PurpleAir station-observed smoke PM_2.5_ within the 2.0° × 2.5° grid box for each day. Although this is lower than the within-block spatial variations accounted for by the estimates of Childs et al.^17^ (within-block *R*^2^ = 0.32), it suggests that our model can explain some spatial variations of fire-sourced PM_2.5_ at a resolution higher than the resolution of GEOS-Chem simulations, after downscaling of the GEOS-Chem outputs, machine learning calibration and including meteorological data inputs at 0.25° × 0.25° spatial resolution.

### Validation against wildfire events

**Extended Data Fig. 5:**
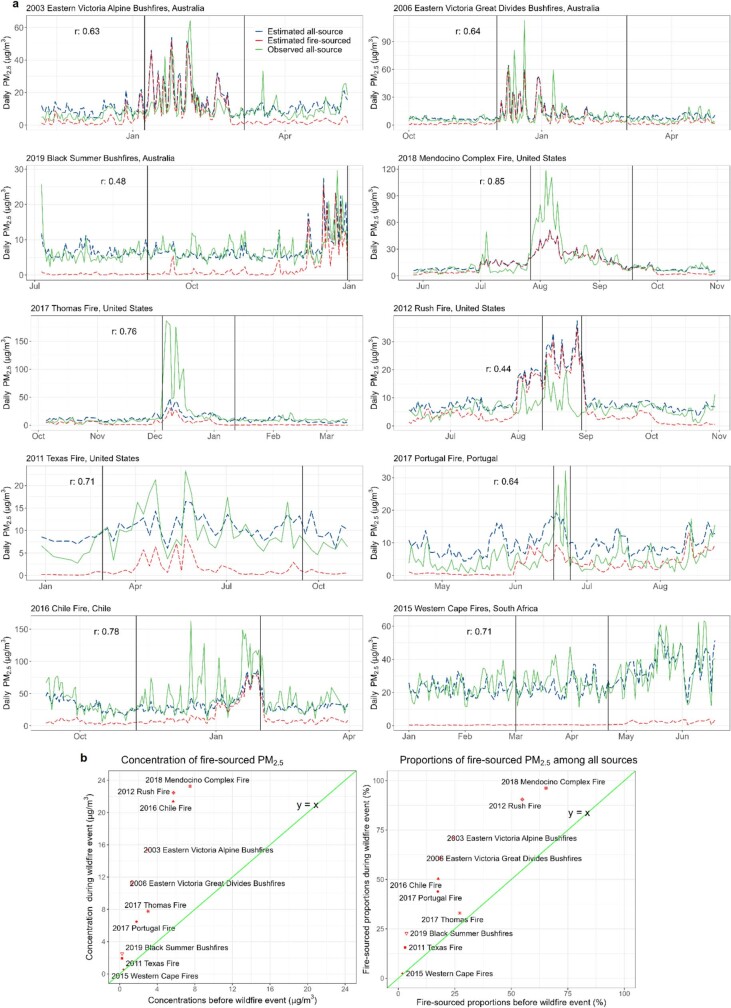
Validation of all-source and fire-sourced PM_2.5_ against large wildfire events. **a**, Line plots showing the time-series trend of daily all-sourced and fire-sourced PM_2.5_ concentrations before, during, and after ten large wildfire events. The two vertical solid lines represent the start date and the end date of the wildfire event. The end date of the 2019 Black Summer Bushfires was not the real end date of the bushfire event, but the last date that our model can cover. **b**, Scatter plots comparing the average daily fire-sourced PM_2.5_ concentrations (left) and the proportions of fire-sourced PM_2.5_ among all-source PM_2.5_ (right) during and before the ten selected large wildfire events. The period before each wildfire event was defined as 60 days before the wildfire event start date.

**Extended Data Fig. 6:**
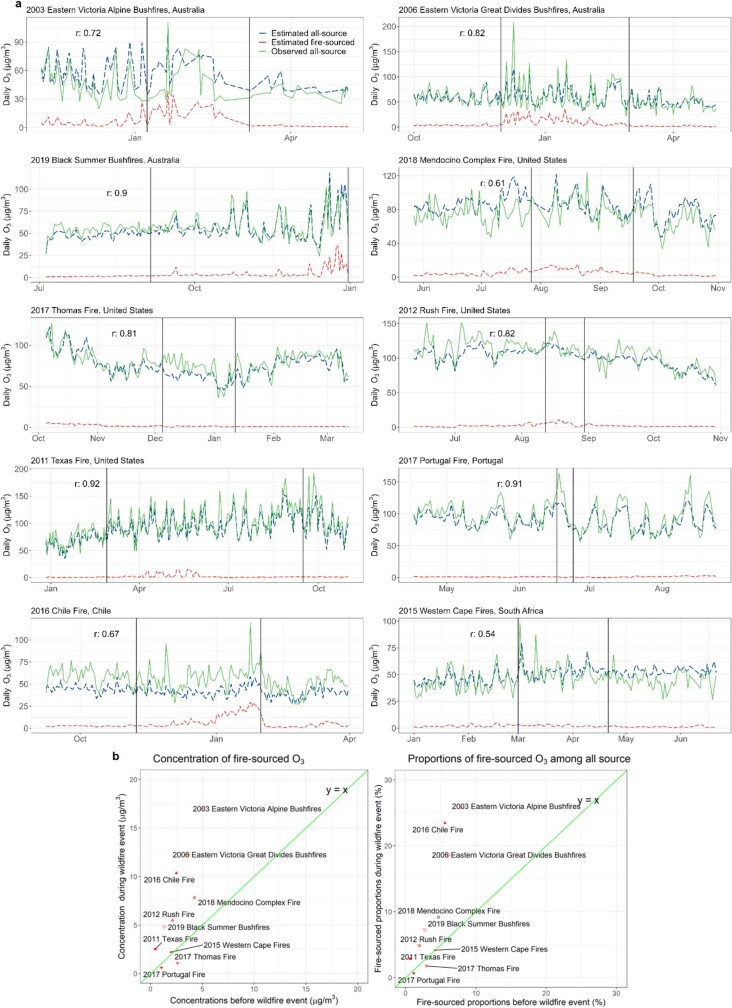
Validation of all-source and fire-sourced O_3_ against large wildfire events. **a**, Line plots showing the time-series trend of daily all-sourced and fire-sourced O_3_ concentrations before, during, and after ten large wildfire events. The two vertical solid lines represent the start date and the end date of the wildfire event. The end date of the 2019 Black Summer Bushfires was not the real end date of the bushfire event, but the last date that our model can cover. **b**, Scatter plots comparing the average daily fire-sourced O_3_ concentrations (left) and the proportions of fire-sourced O_3_ among all-source O_3_ (right) during and before the ten selected large wildfire events. The period before each wildfire event was defined as 60 days before the wildfire event start date.

As detailed in the Supplementary Information, we chose ten large wildfire events in Australia, the USA, Chile, Portugal and South Africa to validate our estimated all-source and fire-sourced PM_2.5_ and O_3_ (Supplementary Table 3). According to the results (Extended Data Figs. 5 and 6), during the wildfire event and up to 60 days before and after the event, the observed daily all-source PM_2.5_ or O_3_ from the most affected monitoring stations (that is, a nearby station showing the largest increase in observed concentrations during the wildfire event, compared with the pre-wildfire period, for each event) showed moderate to strong correlation with our estimated daily all-source PM_2.5_ (*r*, 0.44–0.85; pooled *R*^2^ across wildfire events = 0.64) and O_3_ (*r*, 0.54–0.92; pooled *R*^2^ = 0.78), based on the model trained in the data excluding nearby stations. Furthermore, these was an increase in the estimated concentrations and proportions (among all sources) of fire-sourced PM_2.5_ during all the selected wildfire events, compared with the pre-wildfire period (Extended Data Fig. 5b). There was also an increase in the estimated concentrations and proportions (among all sources) of fire-sourced O_3_ during eight of the ten selected wildfire events (Extended Data Fig. 6b); the two exceptions in which there was decreased fire-sourced O_3_ during the wildfire period could be explained by the uncertain impacts of wildfires on ambient O_3_ (Discussion). Overall, the results indicate that our models can reasonably capture the wildfires’ contribution to the all-source and fire-sourced PM_2.5_ and O_3_.

### Mapping population exposure

The estimated global fire-sourced PM_2.5_ and O_3_ during the period 2000–2019 were linked with global population distribution data to map the global population exposure to daily LFS air pollution. The population exposure was measured by four metrics: (1) population-weighted average fire-sourced PM_2.5_ and O_3_ concentrations (that is, average of all grids weighted by population count of each grid); (2) annual number of person-days exposed to SFAP, with 1 person-day referring to one person exposed to 1 day of SFAP; (3) annual average number of days per person exposed to SFAP, equal to the metric 2 divided by total population size; and (4) annual total number of people exposed to at least 1 day of SFAP.

A day with SFAP should consist of at least one of the following scenarios: (1) the daily average PM_2.5_ (all-source PM_2.5_) exceeded the WHO’s 2021 daily guideline value (15 µg m^−3^), and fire-sourced PM_2.5_ accounted for at least 50% of the daily all-source PM_2.5_, and (2) the daily maximum 8 h O_3_ (all-source O_3_) exceeded the WHO’s 2021 daily guideline value (100 µg m^−3^), and fire-sourced O_3_ accounted for at least 50% of the daily all-source O_3_.

All descriptive analyses were at global scale, and by continent (Africa, Asia, Europe, North America, South America and Oceania), country or territory, HDI group and income group, for each year from 2000 to 2019. Our analyses included 206 countries or territories covered by the ERA5 land grids. We tested the long-term trend of each metric using linear regressions, with the annual metrics during the period 2000–2019 as the dependent variable and year (numeric) as the only predictor.

### Sensitivity analyses

In our primary analyses, we used the GFED4.1s as the fire emission inventory of the GEOS-Chem simulations. However, previous studies in North America found that chemical transport model simulations based on different fire emission inventories generated very different estimates of fire-sourced PM_2.5_ and O_3_^70,71^. Therefore, apart from the GFED4.1s^56^, we also collected data from three other widely used global fire emission inventories: the Fire INventory from the National Center for Atmospheric Research (NCAR) v.1.6 (FINN1.6)^72^, the Quick Fire Emission Dataset v.2.5 (QFED2.5)^73^ and the Global Fire Assimilation System v.1.2 (GFAS1.2)^74^. Each inventory has its own advantages and disadvantages; thus we cannot decide which one is best without validation against real-world observations, although the GFED4.1s is the one with the best data availability (Supplementary Table 4).

Because a previous study suggested that the largest difference of population-weighted fire-sourced PM_2.5_ estimates in North America between four different fire emission inventories was observed in 2012^70^, we ran GEOS-Chem simulations for 2012 using GFED, FINN, QFED and GFAS separately, and performed the aforementioned machine learning calibrations against air quality station data. To ensure comparability, we used the same station and linked predictor data that were available in 2012 in model training and tenfold spatial CV for all four fire emission inventories. We also validated the estimated fire-sourced PM_2.5_ based on different inventories against the station-observed smoke PM_2.5_ in 2012 provided by Childs et al.^17^.

**Extended Data Fig. 7:**
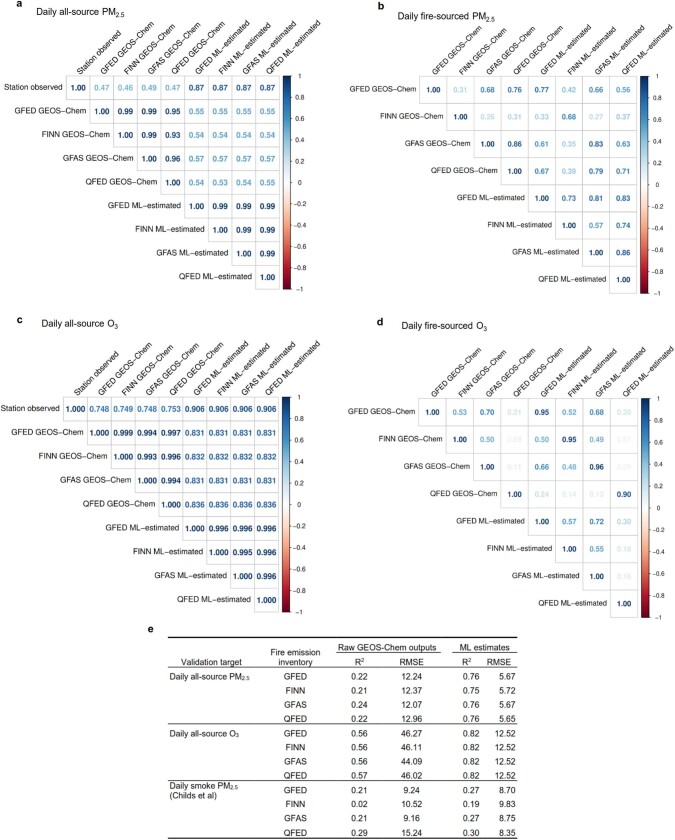
Comparisons between four fire emission inventories. **a**, Correlation matrix showing the Pearson correlations between station observed all-source daily PM_2.5_, and GEOS-Chem simulated and machine learning (ML) estimated all-source PM_2.5_ based on different fire emission inventories in 2012. **b**, Correlation matrix showing the Pearson correlations between GEOS-Chem simulated and ML estimated fire-sourced PM_2.5_ based on different fire emission inventories in 2012. **c**, Correlation matrix showing the Pearson correlations between station observed all-source daily O_3_, and GEOS-Chem simulated and ML estimated all-source O_3_ based on different fire emission inventories in 2012. **d**, Correlation matrix showing the Pearson correlations between GEOS-Chem simulated and ML estimated fire-sourced O_3_ based on different fire emission inventories in 2012. **e**, Table showing the performance of GEOS-Chem outputs and machine learning estimates based on different fire emission inventories in 2012 in validation against station observed daily all-source PM_2.5_, all-source O_3_ and smoke PM_2.5_. RMSE, root mean squared error, in µg/m^3^. For all the panels in this figure, the ML estimates were based on the ML model trained in out-of-sample stations (according to the 10-fold spatial cross-validation). For each specific fire emission inventory (e.g., FINN), the ML predictors include corresponding GEOS-Chem simulated all-source PM_2.5_ or O_3_ (e.g., the FINN GEOS-Chem all-source PM_2.5_) and other predictors mentioned in equation 1 (or 2) in the Methods section.

According to the validation results, the estimated all-source PM_2.5_ and O_3_ based on different fire emission inventories were highly consistent with each other (*r*, 0.99 or above), and they showed very similar accuracy in validation against station observations (spatial tenfold CV *R*^2^ for different inventories, 0.75–0.76 for PM_2.5_ and all 0.82 for O_3_; RMSE, 5.65–5.72 µg m^−3^ for PM_2.5_ and all 12.52 µg m^−3^ for O_3_) (Extended Data Fig. 7). When validated against the station-observed smoke PM_2.5_, the GFED-, GFAS- and QFED-based fire-sourced PM_2.5_ values showed similar accuracy (spatial tenfold CV *R*^2^, 0.27–0.30; RMSE, 8.35–8.75 µg m^−3^), whereas the FINN-based estimates showed the least accuracy (*R*^2^ = 0.19, RMSE = 9.83 µg m^−3^). The GFED-based fire-sourced PM_2.5_ showed good agreement with FINN-, GFAS- and QFED-based estimates (*r*, 0.73, 0.81 and 0.83, respectively). The GFED-based fire-sourced O_3_ showed moderate agreement with FINN-based estimates (*r*, 0.57), good agreement with GFAS-based estimates (*r*, 0.72) and poor agreement with QFED-based estimates (*r*, 0.30); the QFED-based fire-sourced O_3_ showed even poorer agreement with FINN- and GFAS-based estimates (*r*, 0.18 and 0.16, respectively).

**Extended Data Fig. 8:**
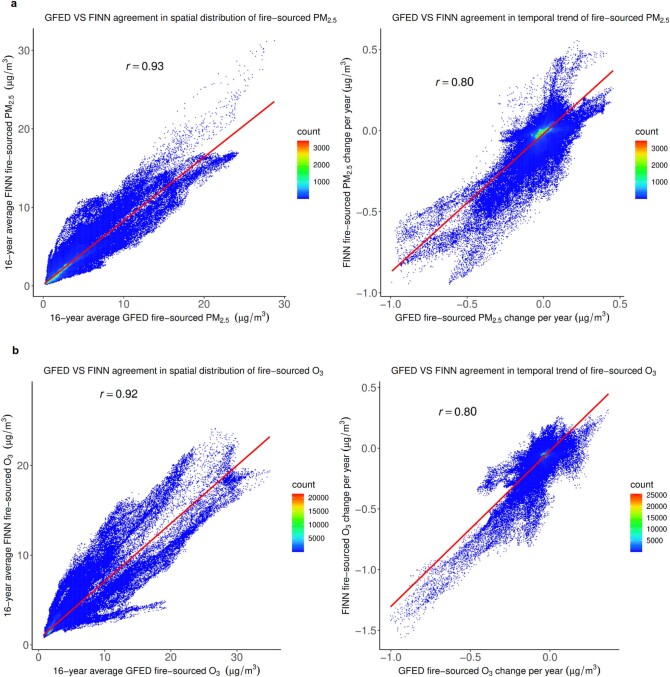
Overall agreements between GFED- and FINN-based estimates in global spatial distribution and temporal trends. **a**, Density scatter plots showing the agreements between GFED and FINN in the overall spatial distribution (left) and temporal trend (right) of estimated fire-sourced PM_2.5_ during 2002–2017. **b**, Density scatter plots showing the agreements between GFED and FINN in the overall spatial distribution (left) and temporal trend (right) of estimated fire-sourced O_3_ during 2002–2017. In panel a and b, the daily fire-sourced PM_2.5_ or O_3_ were estimated using the GEOS-Chem simulations plus machine learning model calibration approach, as detailed in the Methods section. FINN data were only available from 2002 to 2017, so here the 16-year average and the change per year were estimated for this period. The count refers to the count of 0.25° × 0.25° land grids, and all the 394,899 grids across the global land were included in the analyses. For each grid, the temporal trend from 2002 to 2017 was fitted using all annual concentrations during the period with a linear regression.

**Extended Data Fig. 9:**
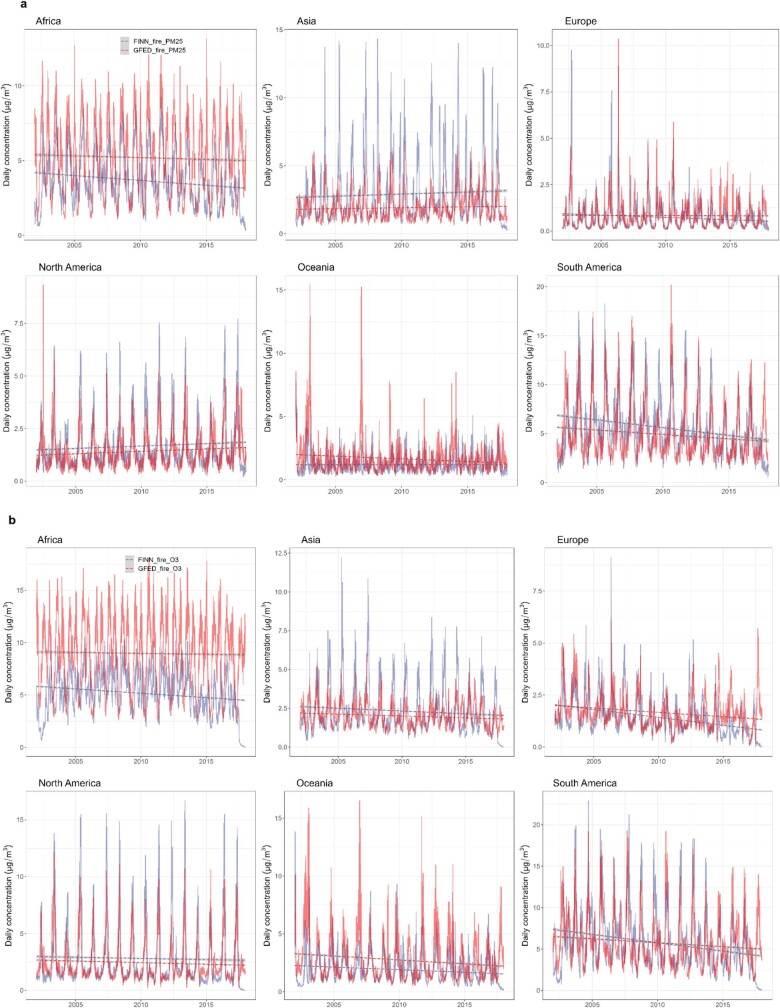
Continent-specific agreements between GFED- and FINN-based estimates of daily fire-sourced PM_2.5_ and O_3_. **a**, Line plots showing the agreements between GFED and FINN in the continent-specific daily population-weighted average fire-sourced PM_2.5_ estimates during 2002–2017. **b**, Line plots showing the agreements between GFED and FINN in the continent-specific daily population-weighted average fire-sourced O_3_ estimates during 2002–2017. In both panels, the fire-sourced PM_2.5_ or O_3_ were estimated using the GEOS-Chem simulations plus machine learning model calibration approach, as detailed in the Methods section. The dashed lines refer to trend lines fitted using linear regressions.

Because FINN showed least agreement with the GFED-based estimates of fire-sourced PM_2.5_ (the main contributor to SFAP), we performed sensitivity analyses by running GEOS-Chem simulations based on FINN for all its available years (2002–2017), and generated the daily FINN-based estimates of fire-sourced PM_2.5_ and O_3_ at 0.25° × 0.25° spatial resolution using the same machine learning calibration procedures as in our primary analyses. Compared with GFED-based estimates during the period 2002–2017, the FINN-based estimates of fire-sourced PM_2.5_ and O_3_ showed very similar spatial distribution (GFED versus FINN agreement in grid-specific 16-year average concentrations, *r* = 0.93 for fire-sourced PM_2.5_, *r* = 0.92 for fire-sourced O_3_), temporal trends (GFED versus FINN agreement in grid-specific change in concentrations per year, *r* = 0.80 for both fire-sourced PM_2.5_ and O_3_), continent-specific long-term trends and seasonal patterns (Extended Data Figs. 8 and 9).

### Uncertainties of our estimates

There were some uncertainties or potential errors in the processes of estimating fire-sourced PM_2.5_ and O_3_. First, the GFED4.1s used for GEOS-Chem simulations has some uncertainties and limitations, such as uncertainties in the emission factor and the estimation of burned areas based on satellite images^56^. Studies suggest that GEOS-Chem simulations based on different fire emission inventories may generate very different estimates of fire-sourced PM_2.5_ in North America^70,71^. However, according to our validation results (Extended Data Fig. 7 and Supplementary Table 4), the GFED4.1s was the best inventory of the four widely used inventories considering both accuracy (that is, agreement with ground station observations and the smoke PM_2.5_of Childs et al.^17^) and data availability, and it is also the most widely used one at present^70^. Our results also suggest that the estimates of all-source and fire-sourced PM_2.5_ and O_3_ based on three alternative inventories were mostly highly consistent with GFED-based estimates, and the consistency improved after machine learning calibrations (Extended Data Fig. 7). Furthermore, even based on FINN (the inventory that showed the least agreement with GFED-based estimates of fire-sourced PM_2.5_), the generated estimates of fire-sourced PM_2.5_ and O_3_ showed spatial distribution, temporal trends and seasonal patterns that were very similar to GFED-based estimates (Extended Data Figs. 8 and 9). Therefore, our assessment of population exposure to fire-sourced air pollution was robust against the choice of fire emission inventory.

Second, our GEOS-Chem simulations did not account for plume rise and assumed that all fire emissions were emitted at the surface, because there are large uncertainties in the fire plume height data^75,76^, and a recent study found that including the fire plume rise did not always improve the accuracy of simulated PM_2.5_ and O_3_^77^. GEOS-Chem simulations without considering plume rise can overestimate the contribution of fire emissions to surface PM_2.5_ and O_3_ in fire source regions while underestimating the impacts of fire emissions in regions downwind from the fire source^75,77^. Given that fire source regions (for example, wildlands or agricultural lands) tend to have smaller population densities than other regions, our GEOS-Chem approach is likely to cause an underestimation of global population exposure to fire-sourced air pollution. Further studies are warranted to quantify and correct the bias caused by omitting plume rise.

Third, the GEOS-Chem was run at a coarse spatial resolution (2.0° × 2.5°), which may cause errors in population exposure assessment at high spatial resolution. However, we have performed downscaling of the GEOS-Chem and added higher-resolution meteorological data as extra predictors in the machine learning model. The validation against observed smoke PM_2.5_ in PurpleAir stations suggested that our estimated fire-sourced PM_2.5_ can explain about 10% of spatial variations of the observed smoke PM_2.5_ within the large 2.0° × 2.5° grid box (Extended Data Fig. 4b), which was a big improvement compared with the raw GEOS-Chem outputs. Moreover, there was almost no correlation between grid-specific population counts and the annual fire-sourced PM_2.5_ (*r* = −0.02) and O_3_ (*r* = 0.001) concentrations in our data, suggesting that the bias in concentration caused by coarse-resolution of GEOS-Chem tend to be distributed to 0.25° × 0.25° grid boxes with high or low population counts randomly and cause random errors, rather than systematic errors of population exposure assessment. Nevertheless, cautions should be taken if our data are used to perform individual-level exposure assessment in epidemiological studies.

Finally, the machine learning models were trained against station observations dominated by several regions (Europe, the USA and China), which may not apply to regions with few or no stations. However, according to our spatial-cluster CV that mimics this situation, our models showed good accuracy in predicting observations far away from the training stations (overall *R*^2^, 0.69 for PM_2.5_ and 0.67 for O_3_), and the accuracy was still much higher than the raw GEOS-Chem outputs even in continents (Africa, South America and Oceania) with a small number of stations (Extended Data Fig. 4a), suggesting that our trained machine learning model can also add accuracy to the GEOS-Chem in regions with limited or no training stations.

We performed the downscaling of GEOS-Chem outputs using ArcGIS desktop (v.10.1); all other data analyses were performed using R software (v.4.0.2).

## Online content

Any methods, additional references, Nature Portfolio reporting summaries, source data, extended data, supplementary information, acknowledgements, peer review information; details of author contributions and competing interests; and statements of data and code availability are available at 10.1038/s41586-023-06398-6.

### Supplementary information

Supplementary Information Supplementary methods, tables and additional references. Supplementary methods: this section contains more detailed information about the process of cleaning the monitoring station data, and the validation of the estimated all-source and fire-sourced PM_2.5_ and O_3_ against large wildfire events. Supplementary tables: this section contains four tables: two tables describing the global and country-specific statistics of the daily PM_2.5_ and O_3_ data from monitoring stations, one table showing the basic information of ten selected large wildfire events used for validation and one table describing the four commonly used global fire emission inventories.

Peer Review File

### Source data

Source Data Fig. 1

Source Data Fig. 2

Source Data Fig. 3

Source Data Fig. 4

## Figures and Tables

**Extended Data Table 1:**
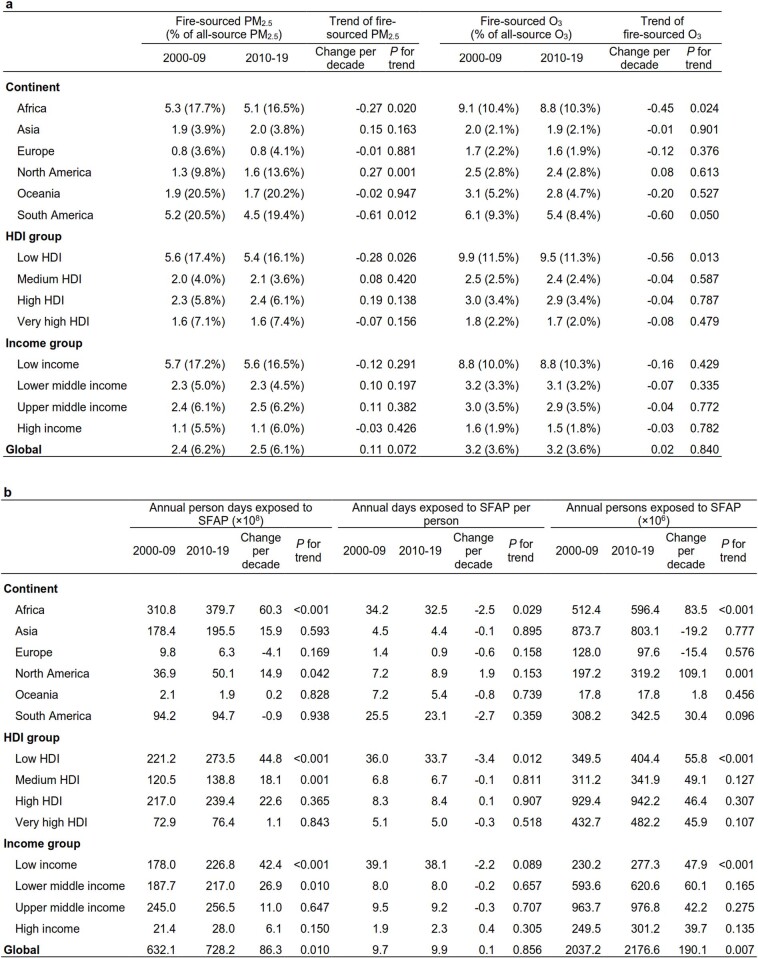
Tables of decadal average exposures for the globe and main subgroups

**a**, Table showing the global annual population-weighted average landscape fire-sourced PM_2.5_ and O_3_ during 2000–2019, by continent, human development index (HDI) group and income group. The units of PM_2.5_ and O_3_ concentrations are both µg/m^3^. **b**, Table showing the population exposure to substantial fire-sourced air pollution (SFAP) during 2000–2019, by continent, HDI group and income group.

## Data Availability

Data of air quality stations are available for free or with certain conditions from the US Environmental Protection Agency (https://aqs.epa.gov/aqsweb/airdata/download_files.html), the China National Environmental Monitoring Centre (http://www.cnemc.cn/en/), the European Environment Agency (https://www.eea.europa.eu/data-and-maps/data/aqereporting-9), the Australian National Air Pollution Monitor Database (http://cardat.github.io/), New Zealand’s Environment Canterbury (http://data.ecan.govt.nz/Catalogue/Method?MethodId=98), the Chilean National Air Quality Information System (https://sinca.mma.gob.cl/index.php/region/index/id/II), the South African Air Quality Information System (https://saaqis.environment.gov.za/), AirQo (https://www.airqo.net/) and OpenAQ (https://openaq.org/). The cleaned air quality station data used for this study were deposited at 10.17605/OSF.IO/DN7YA. Data of weather predictors are open access and are available from https://cds.climate.copernicus.eu/cdsapp#!/dataset/reanalysis-era5-single-levels?tab=overview. Population exposure estimates globally, for different continents, HDI and income groups, and for 206 countries and territories were shared on https://github.com/Rongbin553/wildfire_population. The GEOS-Chem simulation outputs and estimated all-source and fire-sourced air pollution data are available from the corresponding authors on request, and will be made open access at 10.17605/OSF.IO/DN7YA after the paper is published. Source data are provided with this paper.

## Code availability

Analysis codes are available from the corresponding authors on request, and will be shared on https://github.com/Rongbin553/wildfire_population.
