# Supplementary material for: Global population exposure to landscape fire air pollution from 2000 to 2019
Source: Nature. 2023 Sep 20;621(7979):521–9. doi: 10.1038/s41586-023-06398-6 (PMC10511322; doi:10.1038/s41586-023-06398-6)
Supplement: Supplementary file 1 — Supplementary methods, tables and additional references. Supplementary methods: this section contains more detailed information about the process of cleaning the monitoring station data, and the validation of the estimated all-source and fire-sourced PM2.5 and O3 against large wildfire events. Supplementary tables: this section contains four tables: two tables describing the global and country-specific statistics of the daily PM2.5 and O3 data from monitoring stations, one table showing the basic information of ten selected large wildfire events used for validation and one table describing the four commonly used global fire emission inventories. [file 41586_2023_6398_MOESM1_ESM.docx]

**Supplementary Methods**

**Cleaning and quality control of** **monitoring station data**

Daily average PM_2.5_ was calculated by averaging all available hourly observations according to local time, and we only kept daily observations calculated from at least 18 valid hourly observations (i.e., ≥75% of 24 hours). Daily maximum 8-h O_3_ were calculated in two steps: 1) calculating moving average 8-h O_3_ (i.e., average of current hour and next 7 hours) based on hourly observations, where a valid 8-h O_3_ was calculated from at least 7 hourly observations after excluding missing and negative records; and 2) choosing the maximum 8-h O_3_ for days with at least 18 valid 8-h O_3_ records within that day.

After excluding invalid observations (e.g., observations < 0, missing values, duplicated and unreasonable extreme observations), we kept 9,528,179 daily average PM_2.5_ observations of 5,661 stations from 73 countries and territories, and 21,097,834 daily 8-h maximum O_3_ observations of 6,851 stations from 58 countries and territories (Supplementary Table 1**-**2, Extended Data Figure 3).

**Validation of the estimated all-source and fire-sourced PM_2.5_ and O_3_ against large wildfire events**

We chose ten large wildfire events in Australia, US, Portugal, Chile and South Africa to validate whether our estimated all-source and fire-sourced PM_2.5_ and O_3_ can reasonably capture the impacts of wildfire events (Supplementary Table 3) ^78-87^. These large wildfire events were searched from Wikipedia (<https://en.wikipedia.org/wiki/List_of_wildfires>) and selected based on the accuracy of the wildfire event information (e.g., location, fire start and end date based on a careful check of the information from multiple sources) and the availability of monitoring station data in surrounding areas.

For each wildfire event, we selected the five nearest stations. Among the five stations, we chose the station that experienced the most significant increase in observed PM_2.5_ (or O_3_) during the wildfire event compared with the pre-wildfire period, i.e., to identify the most affected station, because the air quality of some nearby stations may not necessarily be affected by the wildfires due to some reasons (e.g., wind direction, topography). Then we trained an ML model without the data of all the five nearest stations (i.e., all nearby stations left out in model training to avoid overfitting), the ML model estimates were then validated against the observations of the most affected station in a pre-defined period (including the wildfire period, and 60 days before and after the wildfire period).

There were two key components in our validation. First, whether the model can estimate the all-source daily PM_2.5_ and O_3_ accurately during the pre-defined period, as evidenced by the agreement between model estimates and station observations. Second, whether the estimated fire-sourced PM_2.5_ and O_3_ showed good consistency with the wildfire events, as evidenced by an increase in concentrations and proportions of fire contribution to all-source concentrations during wildfire events compared with pre-wildfire period (defined as 60 days before the wildfire start date).

**Supplementary Tables**

| **Supplementary Table 1.** Statistics of the ground measured daily PM_2.5_ data used in model training | | | | | |
| --- | --- | --- | --- | --- | --- |
| Country/territory | No. of stations | Year | Daily average PM_2.5_ (µg/m^3^) | | |
|  |  |  | min-max | mean±SD | median (IQR) |
| Afghanistan | 2 | 2019-2019 | 14.3-264.1 | 106.7±68.6 | 79.0 (55.5, 154.2) |
| Albania | 6 | 2011-2019 | 1.1-189.0 | 18.8±18.8 | 12.4 (7.5, 23.2) |
| Algeria | 52 | 2019-2019 | 6.7-195.1 | 44.4±21.3 | 40.5 (28.9, 55.0) |
| Argentina | 1 | 2014-2015 | 2.4-28.2 | 9.6±4.6 | 8.5 (6.9, 11.3) |
| Australia | 167 | 2000-2019 | 0.1-267.3 | 7.1±7.1 | 5.8 (3.6, 8.7) |
| Bahrain | 1 | 2016-2019 | 6.9-235.5 | 54.5±25.4 | 49.6 (37.8, 66.1) |
| Bangladesh | 3 | 2015-2019 | 3.2-267.0 | 80.6±60.2 | 56.5 (34.4, 121.4) |
| Belgium | 71 | 2005-2019 | 0.0-250.6 | 13.8±11.5 | 10.6 (6.4, 18.0) |
| Bosnia & Herzegovina | 7 | 2004-2019 | 2.0-264.2 | 46.3±41.2 | 31.2 (19.3, 58.4) |
| Brazil | 21 | 2017-2017 | 3.5-79.2 | 23.5±10.8 | 22.0 (15.3, 30.4) |
| Canada | 126 | 2016-2019 | 0.1-202.3 | 6.6±5.6 | 5.4 (3.7, 8.0) |
| Chile | 39 | 2000-2019 | 2.0-267.5 | 51.5±41.2 | 38.9 (23.8, 66.0) |
| China mainland | 1624 | 2013-2019 | 1.0-267.5 | 45.2±35.1 | 35.4 (21.7, 57.2) |
| Colombia | 29 | 2016-2019 | 2.1-153.2 | 19.7±9.0 | 18.1 (13.6, 24.0) |
| Costa Rica | 1 | 2019-2019 | 3.3-12.7 | 7.4±2.0 | 7.2 (5.9, 8.4) |
| Croatia | 7 | 2013-2019 | 0.0-182.2 | 13.6±14.8 | 9.1 (5.3, 16.0) |
| CuraÃ§ao | 1 | 2018-2019 | 4.5-37.8 | 9.1±4.2 | 8.1 (6.5, 10.4) |
| Czechia | 70 | 2004-2019 | 1.0-266.8 | 20.7±18.2 | 15.6 (10.0, 24.9) |
| Denmark | 3 | 2004-2019 | 0.0-108.8 | 11.5±5.9 | 10.0 (7.9, 13.4) |
| Ethiopia | 2 | 2016-2019 | 5.4-123.0 | 24.1±11.0 | 21.3 (16.2, 29.9) |
| Finland | 21 | 2001-2019 | 0.0-74.8 | 6.9±4.4 | 5.8 (4.0, 8.5) |
| France | 202 | 2011-2019 | 0.0-129.4 | 12.6±9.8 | 9.8 (6.2, 15.7) |
| Germany | 190 | 2003-2019 | 0.0-171.7 | 13.7±10.3 | 10.9 (7.0, 17.0) |
| Greece | 12 | 2016-2019 | 1.0-131.6 | 16.6±9.7 | 14.2 (10.6, 19.5) |
| Guatemala | 2 | 2019-2019 | 5.2-32.8 | 14.0±6.8 | 12.3 (8.5, 18.3) |
| Hong Kong SAR China | 16 | 2018-2019 | 1.1-118.2 | 20.3±11.1 | 18.5 (12.1, 26.1) |
| Iceland | 11 | 2002-2019 | 0.0-242.7 | 7.3±11.7 | 4.5 (3.0, 7.4) |
| India | 11 | 2013-2019 | 1.4-266.7 | 64.3±53.2 | 46.4 (25.9, 85.0) |
| Indonesia | 5 | 2014-2019 | 0.7-185.1 | 41.3±18.3 | 40.4 (27.6, 53.4) |
| Iraq | 2 | 2019-2019 | 9.9-136.5 | 41.8±24.3 | 34.0 (25.5, 52.4) |
| Israel | 1 | 2015-2015 | 4.5-34.8 | 13.6±5.7 | 12.8 (9.8, 16.2) |
| Italy | 85 | 2013-2019 | 0.0-167.0 | 15.6±11.9 | 12.1 (8.0, 18.8) |
| Jordan | 2 | 2019-2019 | 13.5-55.3 | 27.3±8.9 | 26.5 (20.8, 31.2) |
| Kazakhstan | 1 | 2018-2019 | 2.8-261.7 | 26.4±28.8 | 19.4 (10.1, 28.0) |
| Kenya | 2 | 2018-2018 | 0.1-30.2 | 9.3±6.8 | 8.7 (5.1, 12.4) |
| Kosovo | 13 | 2013-2019 | 0.0-250.4 | 22.8±25.6 | 14.8 (8.5, 26.9) |
| Kuwait | 1 | 2017-2019 | 3.6-259.3 | 49.4±28.0 | 43.2 (33.1, 58.4) |
| Kyrgyzstan | 2 | 2019-2019 | 13.1-209.9 | 38.8±31.7 | 26.5 (20.1, 42.0) |
| Laos | 1 | 2019-2019 | 4.3-107.2 | 31.7±21.8 | 26.0 (13.6, 45.4) |
| Lithuania | 5 | 2007-2019 | 0.0-128.9 | 15.1±11.1 | 12.4 (7.5, 19.3) |
| Luxembourg | 4 | 2012-2019 | 0.8-70.1 | 11.4±7.2 | 9.5 (6.6, 14.1) |
| Madagascar | 1 | 2019-2019 | 17.4-223.8 | 87.9±48.2 | 78.7 (50.1, 121.8) |
| Mexico | 26 | 2016-2019 | 0.4-120.1 | 20.1±10.5 | 18.2 (12.8, 25.1) |
| Mongolia | 2 | 2016-2019 | 2.1-266.0 | 55.9±58.2 | 29.2 (14.6, 77.3) |
| Myanmar (Burma) | 1 | 2019-2019 | 3.1-70.1 | 18.8±16.0 | 11.9 (5.6, 28.3) |
| Nepal | 2 | 2017-2019 | 3.7-173.7 | 49.5±32.7 | 42.6 (21.2, 70.8) |
| Netherlands | 50 | 2013-2019 | 0.9-104.5 | 12.6±9.3 | 9.7 (6.5, 15.8) |
| New Zealand | 9 | 2005-2019 | 0.1-136.3 | 9.1±8.2 | 6.6 (4.6, 10.3) |
| Nigeria | 1 | 2014-2014 | 12.4-40.0 | 21.6±9.3 | 17.8 (14.9, 25.0) |
| North Macedonia | 2 | 2012-2017 | 0.7-265.9 | 41.5±38.1 | 29.2 (19.7, 47.2) |
| Norway | 51 | 2003-2019 | 0.0-109.6 | 8.7±6.0 | 7.3 (5.1, 10.6) |
| Pakistan | 4 | 2019-2019 | 13.1-266.7 | 67.7±49.6 | 52.7 (33.5, 81.7) |
| Peru | 12 | 2016-2019 | 0.8-214.5 | 28.5±15.4 | 26.5 (18.7, 34.2) |
| Philippines | 1 | 2014-2014 | 8.6-34.3 | 17.1±6.1 | 14.2 (13.4, 20.0) |
| Poland | 75 | 2006-2019 | 0.0-266.6 | 24.1±20.5 | 17.8 (11.5, 29.6) |
| Portugal | 24 | 2002-2019 | 0.0-239.5 | 9.7±9.3 | 7.2 (4.0, 12.4) |
| Saudi Arabia | 2 | 2019-2019 | 8.4-52.6 | 30.1±9.0 | 29.4 (23.6, 35.2) |
| Serbia | 1 | 2016-2016 | 2.6-132.1 | 21.9±17.9 | 16.7 (10.6, 26.9) |
| Singapore | 1 | 2015-2015 | 15.0-174.1 | 61.3±36.7 | 47.8 (34.5, 84.8) |
| South Africa | 94 | 2001-2019 | 0.0-264.5 | 24.0±21.0 | 18.6 (10.5, 30.9) |
| Spain | 164 | 2004-2019 | 0.0-247.4 | 9.6±7.0 | 8.0 (5.1, 12.2) |
| Sri Lanka | 2 | 2017-2019 | 5.2-87.1 | 24.9±14.4 | 21.3 (14.0, 32.4) |
| Sweden | 27 | 2002-2019 | 0.0-81.4 | 8.0±6.0 | 6.4 (4.1, 9.9) |
| Taiwan, China | 82 | 2016-2019 | 2.6-99.0 | 20.7±11.9 | 17.8 (11.5, 27.4) |
| Tajikistan | 2 | 2019-2019 | 25.3-164.8 | 72.2±34.1 | 62.5 (48.9, 96.1) |
| Turkey | 40 | 2019-2019 | 0.0-192.2 | 21.9±17.4 | 17.0 (11.3, 26.5) |
| Turkmenistan | 2 | 2019-2019 | 9.3-58.2 | 22.5±9.7 | 19.4 (15.9, 26.3) |
| Uganda | 1 | 2017-2019 | 20.5-163.9 | 57.8±19.0 | 55.2 (44.0, 68.7) |
| United Arab Emirates | 3 | 2017-2019 | 3.5-253.4 | 45.7±24.6 | 43.6 (29.2, 57.5) |
| United Kingdom | 93 | 2000-2019 | 0.0-130.4 | 11.7±8.7 | 9.0 (6.1, 14.1) |
| United States | 2058 | 2000-2019 | 0.0-263.2 | 9.9±7.2 | 8.2 (5.3, 12.6) |
| Uzbekistan | 1 | 2018-2019 | 6.5-147.4 | 43.1±24.0 | 37.4 (26.9, 53.4) |
| Vietnam | 5 | 2015-2019 | 2.4-254.2 | 38.4±26.3 | 31.1 (21.1, 46.2) |
| **Global** | 5661 | 2000-2019 | 0.0-267.5 | 21.8±26.5 | 12.2 (6.8, 25.8) |
| Notes: IQR, interquartile range, referring to 25th percentile to 75th percentile; SD, standard deviation; min-max, minimum to maximum value. | | | | | |

| **Supplementary Table 2.** Statistics of the ground measured daily O_3_ data used in model training | | | | | |
| --- | --- | --- | --- | --- | --- |
| Country/territory | No. of stations | Year | Daily maximum 8-hour O_3_ (µg/m^3^) | | |
|  |  |  | min-max | mean±SD | median (IQR) |
| Albania | 7 | 2012-2019 | 5.5-162.7 | 72.6±27.3 | 73.5 (52.0, 92.2) |
| Andorra | 3 | 2012-2019 | 4.0-163.3 | 83.7±27.5 | 86.1 (68.9, 102.3) |
| Australia | 93 | 2000-2019 | 2.5-210.3 | 54.0±17.4 | 52.0 (43.2, 61.6) |
| Bahrain | 1 | 2017-2019 | 30.0-209.8 | 122.6±40.2 | 125.1 (92.2, 151.6) |
| Belgium | 44 | 2000-2019 | 0.0-210.6 | 61.8±29.4 | 60.3 (43.3, 77.4) |
| Bosnia & Herzegovina | 15 | 2006-2019 | 0.9-210.0 | 68.7±36.8 | 67.0 (39.5, 93.0) |
| Brazil | 51 | 2017-2017 | 6.1-178.0 | 89.0±30.0 | 89.9 (66.6, 111.5) |
| Bulgaria | 20 | 2000-2019 | 0.0-207.9 | 66.9±29.9 | 67.8 (44.6, 88.6) |
| Canada | 153 | 2016-2019 | 2.5-172.0 | 65.0±19.6 | 64.8 (52.0, 77.3) |
| Chile | 42 | 2000-2019 | 0.0-210.7 | 49.8±29.6 | 43.5 (28.5, 66.5) |
| China mainland | 1624 | 2014-2019 | 1.0-210.7 | 74.3±38.5 | 68.9 (45.7, 97.3) |
| Colombia | 9 | 2018-2019 | 3.6-130.5 | 58.5±18.3 | 57.2 (46.8, 69.6) |
| Croatia | 15 | 2006-2019 | 0.0-199.9 | 79.7±30.9 | 81.1 (58.8, 101.8) |
| CuraÃ§ao | 1 | 2018-2019 | 24.7-63.3 | 40.3±7.9 | 39.8 (35.1, 44.9) |
| Cyprus | 3 | 2000-2019 | 0.0-164.9 | 93.4±22.9 | 94.6 (80.2, 109.4) |
| Czechia | 68 | 2000-2019 | 1.0-210.0 | 73.1±30.4 | 71.5 (52.3, 93.5) |
| Denmark | 9 | 2001-2019 | 0.6-189.6 | 66.0±21.4 | 66.5 (52.4, 79.2) |
| Estonia | 9 | 2000-2019 | 2.1-175.8 | 67.1±20.3 | 66.1 (53.4, 80.0) |
| Finland | 24 | 2000-2019 | 1.0-183.5 | 65.9±19.1 | 65.1 (53.1, 78.4) |
| France | 448 | 2000-2019 | 0.0-210.7 | 71.2±29.3 | 70.3 (51.9, 89.1) |
| Germany | 296 | 2000-2019 | 0.0-210.7 | 67.4±31.0 | 65.8 (46.8, 85.8) |
| Gibraltar | 1 | 2005-2019 | 11.3-159.3 | 79.4±20.3 | 78.8 (66.0, 92.5) |
| Greece | 25 | 2000-2019 | 0.0-210.1 | 78.9±35.2 | 78.4 (53.8, 104.0) |
| Hong Kong SAR China | 16 | 2018-2019 | 2.1-210.1 | 73.4±40.4 | 67.6 (42.1, 98.7) |
| Hungary | 18 | 2000-2019 | 0.1-210.2 | 71.3±32.4 | 70.3 (46.3, 95.2) |
| Iceland | 1 | 2004-2013 | 23.6-160.6 | 82.3±17.0 | 82.8 (69.5, 95.1) |
| Ireland | 15 | 2000-2019 | 0.0-202.3 | 64.1±20.4 | 64.5 (49.7, 78.5) |
| Italy | 387 | 2000-2019 | 0.0-210.7 | 76.8±36.8 | 77.8 (51.3, 101.4) |
| Kosovo | 12 | 2013-2019 | 0.0-192.4 | 60.1±30.3 | 59.2 (38.8, 81.8) |
| Latvia | 8 | 2013-2019 | 2.7-154.2 | 66.6±19.3 | 67.2 (54.5, 79.3) |
| Lithuania | 14 | 2002-2019 | 0.1-172.9 | 62.5±22.5 | 60.9 (46.3, 77.1) |
| Luxembourg | 5 | 2004-2019 | 1.3-208.9 | 67.2±29.5 | 65.5 (48.0, 84.0) |
| Malta | 5 | 2004-2019 | 8.3-178.4 | 85.9±20.3 | 85.7 (72.6, 99.2) |
| Mexico | 46 | 2016-2019 | 2.0-210.3 | 104.9±36.2 | 103.5 (78.3, 130.0) |
| Montenegro | 2 | 2012-2019 | 6.4-149.6 | 85.0±23.2 | 87.2 (70.7, 100.9) |
| Myanmar (Burma) | 1 | 2019-2019 | 3.7-58.6 | 20.9±16.2 | 13.0 (8.7, 31.3) |
| Nepal | 2 | 2017-2019 | 5.9-203.2 | 88.8±36.7 | 84.9 (65.3, 115.3) |
| Netherlands | 51 | 2000-2019 | 0.0-210.7 | 61.5±27.0 | 61.0 (44.7, 76.1) |
| New Zealand | 2 | 2002-2017 | 5.6-79.9 | 42.1±9.9 | 42.0 (35.1, 49.5) |
| North Macedonia | 15 | 2003-2019 | 0.8-209.9 | 73.8±36.9 | 72.3 (46.5, 99.5) |
| Norway | 14 | 2000-2019 | 1.1-171.4 | 69.7±18.7 | 70.1 (58.2, 81.8) |
| Peru | 10 | 2016-2018 | 0.6-181.6 | 27.9±15.8 | 27.0 (17.5, 36.2) |
| Poland | 132 | 2000-2019 | 0.0-209.3 | 68.9±28.4 | 67.6 (48.5, 88.2) |
| Portugal | 50 | 2000-2019 | 0.0-210.0 | 73.6±24.7 | 73.0 (57.9, 88.3) |
| Romania | 103 | 2004-2019 | 0.0-209.6 | 60.5±27.5 | 58.8 (39.6, 79.2) |
| Serbia | 10 | 2003-2019 | 0.0-200.1 | 65.0±34.0 | 62.7 (37.9, 91.9) |
| Slovenia | 13 | 2000-2019 | 1.4-209.7 | 78.5±34.2 | 80.4 (54.4, 102.8) |
| South Africa | 103 | 2000-2019 | 0.0-210.6 | 76.7±33.7 | 74.5 (54.0, 98.0) |
| Spain | 467 | 2000-2019 | 0.0-209.0 | 75.9±26.4 | 76.4 (59.0, 93.5) |
| Sweden | 34 | 2000-2019 | 0.1-201.8 | 67.7±19.3 | 66.9 (55.1, 79.6) |
| Switzerland | 39 | 2000-2019 | 0.0-210.6 | 70.4±34.9 | 70.2 (45.5, 93.0) |
| Taiwan, China | 80 | 2016-2019 | 2.6-210.3 | 94.0±32.0 | 92.0 (71.4, 115.6) |
| Thailand | 50 | 2015-2019 | 2.0-210.3 | 76.3±37.9 | 69.5 (47.4, 100.1) |
| Turkey | 50 | 2019-2019 | 1.9-208.6 | 59.1±34.1 | 56.4 (31.1, 85.0) |
| United Arab Emirates | 3 | 2018-2019 | 4.4-203.4 | 80.0±38.7 | 84.4 (55.0, 107.1) |
| United Kingdom | 92 | 2000-2019 | 0.0-209.5 | 60.9±22.1 | 61.5 (47.7, 74.5) |
| United States | 2039 | 2000-2019 | 2.0-210.0 | 82.4±28.5 | 80.5 (62.8, 100.1) |
| Uzbekistan | 1 | 2018-2019 | 3.4-175.9 | 80.3±39.4 | 77.7 (47.7, 109.7) |
| **Global** | 6851 | 2000-2019 | 0.0-210.7 | 75.1±31.3 | 73.5 (54.0, 94.2) |
| Notes: IQR, interquartile range, refer to the 25th percentile to 75th percentile; SD, standard deviation; min-max, minimum to maximum value. | | | | | |

**Supplementary Table 3.** Basic information of ten selected large wildfire events used for validation across the globe

| **Fire name** | **Country** | **Dates** | **Approximate Centroid** | **Information** |
| --- | --- | --- | --- | --- |
| 2003 Eastern Victoria Alpine Bushfires | Australia | 2003-01-08 to 2003-03-08 | lat = -36.8, lon = 146.8 | The Eastern Victoria Alpine Bushfires started on 8 January 2003 and lasted until 8 March 2003, which was the largest fire in Victoria since the Black Friday fires in 1939. There were 87 fires in the North-East and East Gippsland regions, ignited by lightning. The majority of the area burnt in Victoria was public land, 1.19 million hectares of parks and forests, of which Mt Buffalo National Park was one of the hardest hit areas, burned by 81 percent. ^78^ |
| 2006 Eastern Victoria Great Divides Bushfires | Australia | 2006-12-01 to 2007-03-01 | lat = -36.8, lon = 146.4 | The Eastern Victoria Great Divides Bushfires started on 1 Dec 2006 and lasted until March 2007, which were the longest running bushfires in the history of Victoria state, due to lightning strikes. There were over 1 million hectares burned and 255 other buildings damaged. ^79^ |
| 2019 Black Summer Bushfires | Australia | 2019-09-05 to 2019-12-31 (this end date was not the real end date of the bushfire event, but the last date that our model can cover) | lat = -37.8, lon = 144.9 | The Black Summer Bushfires started on 5 Sept 2019 and lasted over half year, from which at least 1 billion wild animals were estimated to die with some species thought to be facing extinction. In Victoria, around 1.4 million hectares have been destroyed and 4 people lost their lives. ^80^ |
| 2018 Mendocino Complex Fire | United States | 2018-07-27 to 2018-09-18 | lat = 39.2, lon = -123.1 | It is California's largest wildfire until 2021. Consisting of two wildfires, the River Fire and Ranch Fire, it was first reported on July 27, 2018, and both fires burned a combined total of 459,123 acres (1,858 km^2^). They were collectively 100% contained on September 18. ^81^ |
| 2017 Thomas Fire | United States | 2017-12-05 to 2018-01-12 | lat = 34.5, lon = -119.1 | It is the largest [wildfire in modern California history](https://en.wikipedia.org/wiki/List_of_California_wildfires) until 2018, ignited in [southern California](https://en.wikipedia.org/wiki/Southern_California) in December 2017 and burned approximately 281,893 acres before being fully contained on January 12, 2018. ^82^ |
| 2012 Rush Fire | United States | 2012-08-12 to 2012-08-30 | lat = 40.7, lon = -120.1 | The second-largest wildfire in California since the accurate fire estimates available until 2017. It started in [Lassen County](https://en.wikipedia.org/wiki/Lassen_County,_California), [California](https://en.wikipedia.org/wiki/California), and eventually spread into [Washoe County](https://en.wikipedia.org/wiki/Washoe_County,_Nevada), [Nevada](https://en.wikipedia.org/wiki/Nevada). The fire consumed a total of 315,577 acres (1,280 km^2^) of sagebrush. ^83^ |
| 2011 Texas Fire | United States | 2011-02-27 to 2011-09-15 | lat = 31.8, lon = -99.2 | The 2011 Texas wildfires were a series of destructive wildfires in Texas that occurred in the 2011 fire season. During 2011 in Texas, around 31,453 fires had burned 4,000,000 acres or 16,190 square kilometres (about double the previous record), 2,947 homes (1,939 of which were destroyed over the Labor Day weekend), and over 2,700 other structures. 47.3% of all acreage burned in the United States in 2011 was burned in Texas. The fires had been particularly severe due to the 2011 Southern US drought that covered the state, and was exacerbated by the unusual convergence of strong winds, unseasonably warm temperatures, and low humidity. ^84^ |
| 2017 Portugal Fire | Portugal | 2017-06-17 to 2017-06-24 | lat = 40.0, lon = -8.2 | An intense heat wave preceded the fires, with many areas of Portugal seeing temperatures in excess of 40 °C (104 °F). During the night of 17–18 June, a total of 156 fires erupted across the country, particularly in mountainous areas 200 km (120 mi) north-northeast of Lisbon. The fires began in the Pedrógão Grande municipality before spreading dramatically causing a firestorm. ^85^ |
| 2016 Chile Fire | Chile | 2016-11-08 to 2017-01-31 | lat = -33.1, lon = -71.6 | Wildfires started in the central provinces of the country in November 2016. By mid-December, 49 wildfires were recorded across the regions of O’Higgins, Maule, Biobío, Valparaíso, Metropolitana. Beginning on 15 January 2017, forest fires ravaged the regions of region Metropolitana, Valparaiso, O´Higgins and Maule, significantly impacting rural sectors and some urban areas. Currently, only a few isolated fires remain. ^86^ |
| 2015 Western Cape Fires | South Africa | 2015-03-01 to 2015-04-21 | lat = -33.99, lon = 19.2 | The 2015 Cape wildfires were a series of wildfires that burned across the Western Cape from February to April 2015. Major fires burned across the Cape Peninsula starting on 1 March in Muizenberg and burning until 9 March, when firefighters extinguished it. The fire resulted in 1 death, 56 injuries, over 6,900 hectares (17,000 acres) of burnt land, and 13 damaged properties, of which 3 were destroyed. The cause of the fire was later determined to be arson. There were also major fires near Wellington and Porteville starting in February, which claimed the lives of 3 firefighters, and a fire in Stellenbosch that caused the evacuation of the University of Stellenbosch and burned over 2,900 hectares (7,200 acres) of land. ^87^ |

.

| **Supplementary Table 4.** Four commonly used global fire emission inventories that can be used to drive GEOS-Chem simulations | | | |
| --- | --- | --- | --- |
| **Fire emission inventory name** | **Latest version** | **Daily fire emission data availability for running GEOS-Chem** | **Citation** |
| Global Fire Emissions Database (GFED) with small fires | 4.1s | 2000-2019 | van der Werf et al, 2017 ^56^ |
| Fire INventory from NCAR (FINN) | 1.6 | 2002-2017 | Wiedinmyer et al 2011 ^72^ |
| Global Fire Assimilation System (GFAS) | 1.2 | 2003-2018 | Kaiser et al 2012 ^74^ |
| Quick Fire Emission Dataset (QFED) | 2.5 | 2000-2017 | Darmenov et al 2016 ^73^ |
| Notes: the last date of the data availability check was performed on 18/03/2023. | | | |

# Additional references

78. Australian Disaster Resilience Knowledge Hub. Bushfire - Alpine Region and North-Eastern Victoria, <<https://knowledge.aidr.org.au/resources/bushfire-alpine-region-and-north-eastern-victoria/>> (2023).

79. Community Bushfire Connection. 2006 Coopers Creek and Great Divide Fires • Community Bushfire Connection, <<https://www.communitybushfireconnection.com.au/history/2006-coopers-creek-great-divide-fires/>> (2023).

80. Government set to revise total number of hectares destroyed during bushfire season, <<https://www.9news.com.au/national/australian-bushfires-17-million-hectares-burnt-more-than-previously-thought/b8249781-5c86-4167-b191-b9f628bdd164>> (2020).

81. BBC News. California wildfires: Eight images that reveal scale of devastation, <<https://www.bbc.com/news/world-us-canada-45135098>> (2018).

82. Thomas Fire Incident Report, <<https://www.fire.ca.gov/incidents/2017/12/4/thomas-fire/>> (2017).

83. InciWeb the Incident Information System. Rush Fire, <<https://web.archive.org/web/20160304083743/http:/inciweb.nwcg.gov/incident/3151/>> (2016).

84. Texas Forest Service. 2011 Texas wildfires: common denominators of home destruction, <<https://wildfiretoday.com/2013/03/06/2011-texas-wildfires-common-denominators-of-home-destruction/>> (2013).

85. Turco M, Jerez S, Augusto S, Tarín-Carrasco P, Ratola N, Jiménez-Guerrero P, et al. Climate drivers of the 2017 devastating fires in Portugal. Sci Rep. 2019 Oct 10;9(1):13886.

86. Chile: Wild Fires - Nov 2016. <<https://reliefweb.int/disaster/wf-2016-000138-chl>> (2016).

87. Malgas N. Firefighters battle to extinguish Bainskloof blaze, <<https://ewn.co.za/2015/04/26/Firefighters-battle-to-extinguish-Bainskloof-blaze>> (2015).
